# A Generalized Relative (*α*, *β*)-Entropy: Geometric Properties and Applications to Robust Statistical Inference

**DOI:** 10.3390/e20050347

**Published:** 2018-05-06

**Authors:** Abhik Ghosh, Ayanendranath Basu

**Affiliations:** Indian Statistical Institute, Kolkata 700108, India

**Keywords:** relative entropy, logarithmic super divergence, robustness, minimum divergence inference, generalized renyi entropy

## Abstract

Entropy and relative entropy measures play a crucial role in mathematical information theory. The relative entropies are also widely used in statistics under the name of divergence measures which link these two fields of science through the minimum divergence principle. Divergence measures are popular among statisticians as many of the corresponding minimum divergence methods lead to robust inference in the presence of outliers in the observed data; examples include the ϕ-divergence, the density power divergence, the logarithmic density power divergence and the recently developed family of logarithmic super divergence (LSD). In this paper, we will present an alternative information theoretic formulation of the LSD measures as a two-parameter generalization of the relative α-entropy, which we refer to as the general (α,β)-entropy. We explore its relation with various other entropies and divergences, which also generates a two-parameter extension of Renyi entropy measure as a by-product. This paper is primarily focused on the geometric properties of the relative (α,β)-entropy or the LSD measures; we prove their continuity and convexity in both the arguments along with an extended Pythagorean relation under a power-transformation of the domain space. We also derive a set of sufficient conditions under which the forward and the reverse projections of the relative (α,β)-entropy exist and are unique. Finally, we briefly discuss the potential applications of the relative (α,β)-entropy or the LSD measures in statistical inference, in particular, for robust parameter estimation and hypothesis testing. Our results on the reverse projection of the relative (α,β)-entropy establish, for the first time, the existence and uniqueness of the minimum LSD estimators. Numerical illustrations are also provided for the problem of estimating the binomial parameter.

## 1. Introduction

Decision making under uncertainty is the backbone of modern information science. The works of C. E. Shannon and the development of his famous entropy measure [1,2,3] represent the early mathematical foundations of information theory. The Shannon entropy and the corresponding relative entropy, commonly known as the Kullback-Leibler divergence (KLD), has helped to link information theory simultaneously with probability [4,5,6,7,8] and statistics [9,10,11,12,13]. If *P* and *Q* are two probability measures on a measurable space (Ω,A) and have absolutely continuous densities *p* and *q*, respectively, with respect to a common dominating σ-finite measure μ, then the Shannon entropy of *P* is defined as

(1) $$\begin{array}{c} E(P)=-\int p\log(p)d\mu , \end{array}$$

and the KLD measure between *P* and *Q* is given by

(2) $$\begin{array}{c} \mathrm{RE}\left(P,Q\right)=\int p\log\left(\frac{p}{q}\right)d\mu . \end{array}$$

In statistics, the minimization of the KLD measure produces the most likely approximation as given by the maximum likelihood principle; the latter, in turn, has a direct equivalence to the (Shannon) entropy maximization criterion in information theory. For example, if Ω is finite and μ is the counting measure, it is easy to see that RE(P,U)=log|Ω|−E(P), where *U* is the uniform measure on Ω. Minimization of this relative entropy, or equivalently maximization of the Shannon entropy, with respect to *P* within a suitable convex set E, generates the most probable distribution for an independent identically distributed finite source having true marginal probability in E with non-informative (uniform) prior probability of guessing [14,15]. In general, with a finite source, RE(P,Q) denotes the penalty in expected compressed length if the compressor assumes a mismatched probability *Q* [16,17]. The corresponding general minimizer of RE(P,Q) given *Q*, namely its forward projection, and other geometric properties of RE(P,Q) are well studied in the literature; see [18,19,20,21,22,23,24,25,26,27,28,29] among others.

Although the maximum entropy or the minimum divergence criterion based on the classical Shannon entropy E(P) and the KLD measure RE(P,Q) is still widely used in major (probabilistic) decision making problems in information science and statistics [30,31,32,33,34,35,36,37,38,39,40,41,42,43], there also exist many different useful generalizations of these quantities to address eminent issues in quantum statistical physics, complex codings, statistical robustness and many other topics of interest. For example, if we consider the standardized cumulant of compression length in place of the expected compression length in Shannon’s theory, the optimum distribution turns out to be the maximizer of a generalization of the Shannon entropy [44,45] which is given by

(3) $$\begin{array}{c} E_{\alpha }\left(P\right)=\frac{1}{1-\alpha }\log\left(\int p^{\alpha }d\mu \right),\;\;\;\;\;\alpha >0,\;\alpha \neq 1 \end{array}$$

provided $p\in L_{\alpha }\left(\mu \right)$, the complete vector space of functions for which the α-th power of their absolute values are μ-integrable. This general entropy functional is popular by the name Renyi entropy of order α [46] and covers many important entropy measures like Hartley entropy at α→0 (for finite source), Shannon entropy at α→1, collision entropy at α=2 and the min-entropy at α→∞. The corresponding Renyi divergence measure is given by

(4) $$\begin{array}{c} D_{\alpha }\left(P,Q\right)=\frac{1}{\alpha -1}\log\left(\int p^{\alpha }q^{1-\alpha }d\mu \right),\;\;\alpha >0,\;\alpha \neq 1, \end{array}$$

whenever $p,q\in L_{\alpha }\left(\mu \right)$ and coincides with the classical KLD measure at α→1. The Renyi entropy and the Renyi divergence are widely used in recent complex physical and statistical problems; see, for example, [47,48,49,50,51,52,53,54,55,56]. Other non-logarithmic extensions of Shannon entropy include the classical *f*-entropies [57], the Tsallis entropy [58] as well as the more recent generalized (α,β,γ)-entropy [59,60] among many others; the corresponding divergences and the minimum divergence criteria are widely used in critical information theoretic and statistical problems; see [57,59,60,61,62,63,64,65,66,67,68,69,70] for details.

We have noted that there is a direct information theoretic connection of KLD to the Shannon entropy under mismatched guessing by minimizing the expected compressed length. However, such a connection does not exist between the Renyi entropy $E_{\alpha }\left(P\right)$ and the Renyi divergence $D_{\alpha }\left(P,Q\right)$ as recently noted by [17,71]. Herein, it has been shown that, for a finite source with marginal distribution *P* and a (prior) mismatched compressor distribution *Q*, the penalty in the normalized cumulant of compression length is not $D_{\alpha }\left(P,Q\right)$; rather it is given by $D_{1/\alpha }\left(P_{\alpha },Q_{\alpha }\right)$ where $P_{\alpha }$ and $Q_{\alpha }$ are defined by

(5) $$\begin{array}{c} \frac{dP_{\alpha }}{d\mu }=p_{\alpha }=\frac{p^{\alpha }}{\int p^{\alpha }d\mu },\;\;\;\frac{dQ_{\alpha }}{d\mu }=q_{\alpha }=\frac{q^{\alpha }}{\int q^{\alpha }d\mu }. \end{array}$$

The new quantity $D_{1/\alpha }\left(P_{\alpha },Q_{\alpha }\right)$ also gives a measure of discrimination (i.e., is a divergence) between the probability distributions *P* and *Q* and coincides with the KLD at α→1. This functional is referred to as the relative α-entropy in the terminology of [72] and has the simpler form

(6) $$\begin{array}{cc} {\mathrm{RE}}_{\alpha }\left(P,Q\right) & :=D_{1/\alpha }\left(P_{\alpha },Q_{\alpha }\right)\\ & \;=\frac{\alpha }{1-\alpha }\log\int pq^{\alpha -1}d\mu -\frac{1}{1-\alpha }\log\int p^{\alpha }d\mu +\log\int q^{\alpha }d\mu ,\;\;\;\;\alpha >0,\;\alpha \neq 1. \end{array}$$

The geometric properties of this relative α-entropy along with its forward and reverse projections have been studied recently [16,73]; see Section 2.1 for some details. This quantity had, however, already been proposed earlier as a statistical divergence, although for α≥1 only, by [74] while developing a robust estimation procedure following the generalized method-of-moments approach of [75]. Later authors referred to the divergence proposed in [74] as the logarithmic density power divergence (LDPD) measure. The advantages of the minimum LDPD estimator in terms of robustness against outliers in data have been studied by, among other, [66,74]. Fujisawa [76], Fujisawa and Eguchi [77] have also used the same divergence measure with γ=(α−1)≥0 in different statistical problems and have referred to it as the γ-divergence. Note that, the formulation in (6) extends the definition of the divergence over the 0<α<1 region as well.

Motivated by the substantial advantages of the minimum LDPD inference in terms of statistical robustness against outlying observations, Maji et al. [78,79] have recently developed a two-parameter generalization of the LDPD family, namely the logarithmic super divergence (LSD) family, given by

(7) $$\begin{array}{c} \begin{array}{c} {\mathrm{LSD}}_{\tau ,\gamma }\left(P,Q\right)=\frac{1}{B}\log\int p^{1+\tau }d\mu -\frac{1+\tau }{AB}\log\int p^{A}q^{B}d\mu +\frac{1}{A}\log\int q^{1+\tau }d\mu ,\\ \mathrm{with}\;\;A=1+\gamma (1-\tau ),B=1+\tau -A,\;\;\tau \geq 0,\gamma \in R. \end{array} \end{array}$$

This rich superfamily of divergences contain many important divergence measures including the LDPD at γ=0 and the Kullback-Leibler divergence at τ=γ→0; this family also contains a transformation of Renyi divergence at τ=0 which has been referred to as the logarithmic power-divergence family by [80]. As shown in [78,79], the statistical inference based on some of the new members of this LSD family, outside the existing ones including the LDPD, provide much better trade-off between the robustness and efficiency of the corresponding minimum divergence estimators.

The statistical benefits of the LSD family over the LDPD family raise a natural question: is it possible to translate this robustness advantage of the LSD family of divergences to the information theoretic context, through the development of a corresponding generalization of the relative α-entropy in (6)? In this paper, we partly answer this question by defining an independent information theoretic generalization of the relative α-entropy measure coinciding with the LSD measure. We will refer to this new generalized relative entropy measure as the “*Relative (α,β)-entropy*” and study its properties for different values of α>0 and β∈R. In particular, this new formulation will extend the scope of the LSD measure for −1<τ<0 as well and generate several interesting new divergence and entropy measures. We also study the geometric properties of all members of the relative (α,β)-entropy family, or equivalently the LSD measures, including their continuity in both the arguments and a Pythagorean-type relation. The related forward projection problem, i.e., the minimization of the relative (α,β)-entropy in its first argument, is also studied extensively.

In summary, the main objective of the present paper is to study the geometric properties of the LSD measure through the new information theoretic or entropic formulation (or the relative (α,β)-entropy). Our results indeed generalize the properties of the relative α-entropy from [16,73]. The specific and significant contributions of the paper can be summarized as follows.

1. We present a two parameter extension of the relative α-entropy measure in (6) motivated by the logarithmic *S*-divergence measures. These divergence measures are known to generate more robust statistical inference compared to the LDPD measures related to the relative α-entropy.
2. In the new formulation of the relative (α,β)-entropy, the LSD measures are linked with several important information theoretic divergences and entropy measures like the ones named after Renyi. A new divergence family is discovered corresponding to α→0 case (properly standardized) for the finite measure cases.
3. As a by-product of our new formulation, we get a new two-parameter generalization of the Renyi entropy measure, which we refer to as the Generalized Renyi entropy (GRE). This opens up a new area of research to examine the detailed properties of GRE and its use in complex problems in statistical physics and information theory. In this paper, we show that this new GRE satisfies the basic entropic characteristics, i.e., it is zero when the argument probability is degenerate and is maximum when the probability is uniform.
4. Here we provide a detailed geometric analysis of the robust LSD measure, or equivalently the relative (α,β)-entropy in our new formulation. In particular, we show their continuity or lower semi-continuity with respect to the first argument depending on the values of the tuning parameters α and β. Also, its lower semi-continuity with respect to the second argument is proved.
5. We also study the convexity of the LSD measures (or the relative (α,β)-entropies) with respect to its argument densities. The relative α-entropy (i.e, the relative (α,β)-entropy at β=1) is known to be quasi-convex [16] only in its first argument. Here, we will show that, for general α>0 and β≠1, the relative (α,β)-entropies are not quasi-convex on the space of densities, but they are always quasi-convex with respect to both the arguments on a suitably (power) transformed space of densities. Such convexity results in the second argument were unavailable in the literature even for the relative α-entropy, which we will introduce in this paper through a transformation of space.
6. Like the relative α-entropy, but unlike the relative entropy in (2), our new relative (α,β)-entropy also does not satisfy the data processing inequalities. However, we prove an extended Pythagorean relation for the relative (α,β)-entropy which makes it reasonable to treat them as “squared distances” and talk about their projections.
7. The forward projection of a relative entropy or a suitable divergence, i.e., their minimization with respect to the first argument, is very important for both statistical physics and information theory. This is indeed equivalent to the maximum entropy principle and is also related to the Gibbs conditioning principle. In this paper, we will examine the conditions under which such a forward projection of the relative (α,β)-entropy (or, LSD) exists and is unique.
8. Finally, for completeness, we briefly present the application of the LSD measure or the relative (α,β)-entropy measure in robust statistical inference in the spirit of [78,79] but now with extended range of tuning parameters. It uses the reverse projection principle; a result on the existence of the minimum LSD functional is first presented with the new formulation of this paper. Numerical illustrations are provided for the binomial model, where we additionally study their properties for the extended tuning parameter range α∈(0,1) as well as for some new divergence families (related to α=0). Brief indications of the potential use of these divergences in testing of statistical hypotheses are also provided.

Although we are primarily discussing the logarithmic entropies like the Renyi entropy and its generalizations in this paper, it is important to point out that non-logarithmic entropies including the f-entropy and the Tsallis entropy are also very useful in several applications with real systems. Recently, several complex physical and social systems have been observed to follow the theory developed from such non-logarithmic, non-additive entropies instead of the classical additive Shannon entropy. In particular, the Tsallis entropy has led to the development of the nonextensive statistical mechanics [61,64] to solve several critical issues in modern physics. Important areas of application include, but certainly are not limited to, the motion of cold atoms in dissipative optical lattices [81,82], the magnetic field fluctuations in the solar wind and related q-triplet [83], the distribution of velocity in driven dissipative dusty plasma [84], spin glass relaxation [85], the interaction of trapped ion with a classical buffer gas [86], different high energy collisional experiments [87,88,89], derivation of the black hole entropy [90], along with water engineering [63], text mining [65] and many others. Therefore, it is also important to investigate the possible generalizations and manipulations of such non-logarithmic entropies both from mathematical and application point of view. However, as our primary interest here is in logarithmic entropies, we have, to keep the focus clear, otherwise avoided the description and development of non-logarithmic entropies in this paper.

Although there are many applications of extended and general non-additive entropy and divergence measures, there are also some criticisms of these non-additive measures that should be kept in mind. It is of course possible to employ such quantities simply as new descriptors of the complexity of systems, but at the same time, it is known that the minimization of a generalized divergence (or maximization of the corresponding entropy) under constraints in order to determine an optimal probability assignment leads to inconsistencies for information measures other than the Kullback-Leibler divergence. See, for instance [91,92,93,94,95,96], among others. So, one needs to be very careful in discriminating the application of the newly introduced entropies and divergence measures for the purposes of inference under given information, from the ones where it is used as a measure of complexity. In this respect, we would like to emphasize that, the main advantage of our two-parameter extended family of LSD or relative (α,β)-entropy measures in parametric statistical inference is in their strong robustness property against possible contamination (generally manifested through outliers) in the sample data. The classical additive Shannon entropy and Kullback-Leibler divergence produce non-robust inference even under a small proportion of data contamination, but the extremely high robustness of the LSD has been investigated in detail, with both theoretical and empirical justifications, by [78,79]; in this respect, we will present some numerical illustrations in Section 5.2. Another important issue could be to decide whether to stop at the two-parameter level for information measures or to extend it to three-parameters, four-parameters, etc. It is not an easy question to answer. However, we have seen that many members of the two-parameter family of LSD measures generate highly robust inference along with a desirable trade-off between efficiency under pure data and robustness under contaminated data. Therefore a two-parameter system appears to work well in practice. Since it is a known principle that one “should not multiply entities beyond necessity”, we will, for the sake of parsimony, restrict ourselves to the second level of generalization for robust statistical inference, at least until there is further convincing evidence that the next higher level of generalization can produce a significant improvement.

## 2. The Relative (α,β)-Entropy Measure

### 2.1. Definition: An Extension of the Relative α-Entropy

In order to motivate the development of our generalized relative (α,β)-entropy measure, let us first briefly describe an alternative formulation of the relative α-entropy following [16]. Consider the mathematical set-up of Section 1 with α>0 and assume that the space $L_{\alpha }\left(\mu \right)$ is equipped with the norm

(8) $$\begin{array}{c} {\left||f|\right|}_{\alpha }=\left\{\begin{array}{ccc} {\left({\int |f|}^{\alpha }d\mu \right)}^{1/\alpha } & \mathrm{if} & \alpha \geq 1,\;\;f\in L_{\alpha }\left(\mu \right),\\ {\int |f|}^{\alpha }d\mu & \mathrm{if} & 0<\alpha <1,\;\;f\in L_{\alpha }\left(\mu \right), \end{array}\right. \end{array}$$

and the corresponding metric $d_{\alpha }\left(g,f\right)=||g-{f||}_{\alpha }$ for $g,f\in L_{\alpha }\left(\mu \right)$. Then, the relative α-entropy between two distributions *P* and *Q* is obtained as a function of the Cressie-Read power divergence measure [97], defined below in (11), between the escort measures $P_{\alpha }$ and $Q_{\alpha }$ defined in (5). Note that the disparity family or the ϕ-divergence family [18,98,99,100,101,102,103] between *P* and *Q* is defined as

(9) $$\begin{array}{c} D_{\phi }\left(P,Q\right)=\int q\phi \left(\frac{p}{q}\right)d\mu , \end{array}$$

for a continuous convex function ϕ on [0,∞) satisfying ϕ(0)=0 and with the usual convention 0ϕ(0/0)=0. We consider the ϕ-function given by

(10) $$\begin{array}{c} \phi \left(u\right)=\phi_{\lambda }\left(u\right)=sign\left(\lambda \left(\lambda +1\right)\right)\left(u^{\lambda +1}-1\right),\;\;\;\;\lambda \in R,u\geq 0, \end{array}$$

with the convention that, for any u>0, $0\phi_{\lambda }\left(u/0\right)=0$ if λ<0 and $0\phi_{\lambda }\left(u/0\right)=\infty$ if λ>0. The corresponding ϕ-divergence has the form

(11) $$\begin{array}{c} D_{\lambda }\left(P,Q\right)=D_{\phi_{\lambda }}\left(P,Q\right)=sign\left(\lambda \left(\lambda +1\right)\right)\int q\left[{\left(\frac{p}{q}\right)}^{\lambda +1}-1\right]d\mu , \end{array}$$

which is just a positive multiple of the Cressie-Read power divergence with the multiplicative constant being |λ(1+λ)|; when this constant is present, the case λ=0 leads to the KLD measure in a limiting sense. Note that, our ϕ-function in (10) differs slightly from the one used by [16] in that we use sign(λ(λ+1)) in place of sign(λ) there; this is to make the divergence in (11) non-negative for all λ∈R ([16] considered only λ>−1) which will be needed to define our generalized relative entropy. Then, given an α>0, [16,17] set $\lambda =\alpha^{-1}-1\left(>-1\right)$ and show that the relative α-entropy of *P* with respect to *Q* can be obtained as

(12) $$\begin{array}{c} {\mathrm{RE}}_{\alpha }\left(P,Q\right)={\mathrm{RE}}_{\alpha }^{\mu }\left(P,Q\right)=\frac{1}{\lambda }\log\left[sign\left(\lambda \right)D_{\lambda }\left(P_{\alpha },Q_{\alpha }\right)+1\right]. \end{array}$$

It is straightforward to see that the above formulation (12) coincides with the definition given in (6). We often suppress the superscript μ whenever the underlying measure is clear from the context; in most applications in information theory and statistics it is either counting measure or the Lebesgue measure depending on whether the distribution is discrete or continuous.

We can now change the tuning parameters in the formulation given by (12) suitably as to arrive at the more general form of the LSD family in (7). For this purpose, let us fix α>0, β∈R and assume that $p,q\in L_{\alpha }\left(\mu \right)$ are the μ-densities of *P* and *Q*, respectively. Instead of considering the re-parametrization $\lambda =\alpha^{-1}-1$ as above, we now consider the two-parameter re-parametrization $\lambda =\beta \alpha^{-1}-1\in R$. Note that, the feasible range of λ, in order to make α>0, now clearly depends on β through $\alpha =\frac{\beta }{1+\lambda }>0$; whenever β>0 we have −1<λ<∞ and if β<0 we need −∞<λ<−1. We have already taken care of this dependence through the modified ϕ function defined in (10) which ensures that $D_{\lambda }\left(\cdot ,\cdot \right)$ is non-negative for all λ∈R. So we can again use the relation as in (12), after suitable standardization due to the additional parameter β, to define a new generalized relative entropy measure as given in the following definition.

**Definition** **1** (Relative (α,β)-entropy)**.** *Given any α>0 and β∈R, put $\lambda =\frac{\beta }{\alpha }-1$ (i.e., $\alpha =\frac{\beta }{1+\lambda }$). Then, the relative (α,β)-entropy of P with respect to Q is defined as*

(13) $$\begin{array}{c} {\mathrm{RE}}_{\alpha ,\beta }\left(P,Q\right)={\mathrm{RE}}_{\alpha ,\beta }^{\mu }\left(P,Q\right)=\frac{1}{\beta \lambda }\log\left[sign\left(\beta \lambda \right)D_{\lambda }\left(P_{\alpha },Q_{\alpha }\right)+1\right]. \end{array}$$

*The cases β=0 and λ=0 (i.e, β=α) are defined in limiting sense; see Equations (15) and (16) below.*

A straightforward simplification gives a simpler form of this new relative (α,β)-entropy which coincides with the LSD measure as follows.

(14) $$\begin{array}{ccc} {\mathrm{RE}}_{\alpha ,\beta }\left(P,Q\right) & = & \frac{1}{\alpha -\beta }\log\int p^{\alpha }d\mu -\frac{\alpha }{\beta (\alpha -\beta )}\log\int p^{\beta }q^{\alpha -\beta }d\mu +\frac{1}{\beta }\log\int q^{\alpha }d\mu ,\\ & = & {\mathrm{LSD}}_{\alpha -1,\frac{\beta -1}{2-\alpha }}\left(P,Q\right). \end{array}$$

Note that, it coincides with the relative α-entropy ${\mathrm{RE}}_{\alpha }\left(P,Q\right)$ at the choice β=1. For the limiting cases, it leads to the forms

(15) $$\begin{array}{ccc} {\mathrm{RE}}_{\alpha ,0}\left(P,Q\right) & = & \frac{\int \log\left(q/p\right)q^{\alpha }d\mu }{\int q^{\alpha }d\mu }+\frac{1}{\alpha }\log\left(\frac{\int p^{\alpha }d\mu }{\int q^{\alpha }d\mu }\right), \end{array}$$

(16) $$\begin{array}{ccc} {\mathrm{RE}}_{\alpha ,\alpha }\left(P,Q\right) & = & \frac{\int \log\left(p/q\right)p^{\alpha }d\mu }{\int p^{\alpha }d\mu }+\frac{1}{\alpha }\log\left(\frac{\int q^{\alpha }d\mu }{\int p^{\alpha }d\mu }\right). \end{array}$$

By the divergence property of $D_{\lambda }\left(\cdot ,\cdot \right)$, all the relative (α,β)-entropies are non-negative and valid statistical divergences. Note that, in view of (14), the formulation (13) extends the scope of LSD measure, defined in (7), for τ∈(−1,0).

**Proposition** **1.** For any α>0 and β∈R, ${\mathrm{RE}}_{\alpha ,\beta }\left(P,Q\right)\geq 0$ for all probability measures P and Q, whenever it is defined. Further, ${\mathrm{RE}}_{\alpha ,\beta }\left(P,Q\right)=0$ if and only in P=Q[μ].

Also, it is important to identify the cases where the relative (α,β)-entropy is not finitely defined, which can be obtained from the definition and convention related to $D_{\lambda }$ divergence; these are summarized in the following proposition.

**Proposition** **2.** *For any α>0, β∈R and distributions P,Q having μ-densities in $L_{\alpha }\left(\mu \right)$, the relative (α,β)-entropy ${\mathrm{RE}}_{\alpha ,\beta }\left(P,Q\right)$ is a finite positive number except for the following three cases:*

*1.*  P is not absolutely continuous with respect to Q and α<β, in which case ${\mathrm{RE}}_{\alpha ,\beta }\left(P,Q\right)=+\infty$.
*2.*  P is mutually singular to Q and α>β, in which case also ${\mathrm{RE}}_{\alpha ,\beta }\left(P,Q\right)=+\infty$.
*3.*  0<β<α and $D_{\lambda }\left(P_{\alpha },Q_{\alpha }\right)\geq 1$, in which case also ${\mathrm{RE}}_{\alpha ,\beta }\left(P,Q\right)$ is undefined.

The above two propositions completely characterize the values and existence of our new relative (α,β)-entropy measure. In the next subsection, we will now explore its relation with other existing entropies and divergence measures; along the way we will get some new ones as by-products of our generalized relative entropy formulation.

### 2.2. Relations with Different Existing or New Entropies and Divergences

The relative (α,β)-entropy measures form a large family containing several existing relative entropies and divergences. Its relation with some popular ones are summarized in the following proposition; the proof is straightforward from definitions and hence omitted.

**Proposition** **3.** *For α>0, β∈R and distributions P, Q, the following results hold (whenever the relevant integrals and divergences are defined finitely, even in limiting sense).*

*1.*  ${\mathrm{RE}}_{1,1}\left(P,Q\right)=\mathrm{RE}\left(P,Q\right)$, the KLD measure.
*2.*  ${\mathrm{RE}}_{\alpha ,1}\left(P,Q\right)={\mathrm{RE}}_{\alpha }\left(P,Q\right)$, the relative α-entropy.
*3.*  ${\mathrm{RE}}_{1,\beta }\left(P,Q\right)=\frac{1}{\beta }D_{\beta }\left(P,Q\right)$, a scaled Renyi divergence, which also coincides with the logarithmic power divergence measure of [80].
*4.*  ${\mathrm{RE}}_{\alpha ,\beta }\left(P,Q\right)=\frac{1}{\beta }D_{\beta /\alpha }\left(P_{\alpha },Q_{\alpha }\right)$, where $P_{\alpha }$ and $Q_{\alpha }$ are as defined in (5).

**Remark** **1.** *Note that, items 3 and 4 in Proposition 3 indicate a possible extension of the Renyi divergence measure over negative values of the tuning parameter β as follows:*

$$\begin{array}{ccc} D_{\beta }^{*}\left(P,Q\right) & = & \frac{1}{\beta }D_{\beta }\left(P,Q\right),\;\;\beta \in R\setminus \left\{0\right\},\;\;\;\;D_{0}^{*}\left(P,Q\right)=\int q\log\left(\frac{q}{p}\right)d\mu . \end{array}$$

*Note that this modified Renyi divergence also coincides with the KLD measure at β=1. Statistical applications of this divergence family have been studied by [80].*

However, not all the members of the family of relative (α,β)-entropies are distinct or symmetric. For example, ${\mathrm{RE}}_{\alpha ,0}\left(P,Q\right)={\mathrm{RE}}_{\alpha ,\alpha }\left(Q,P\right)$ for any α>0. The following proposition characterizes all such identities.

**Proposition** **4.** For α>0, β∈R and distributions P, Q, the relative (α,β)-entropy ${\mathrm{RE}}_{\alpha ,\beta }\left(P,Q\right)$ is symmetric if and only if $\beta =\frac{\alpha }{2}$. In general, we have ${\mathrm{RE}}_{\alpha ,\frac{\alpha }{2}-\gamma }\left(P,Q\right)={\mathrm{RE}}_{\alpha ,\frac{\alpha }{2}+\gamma }\left(Q,P\right)$ for any α>0,γ∈R.

Recall that the KLD measure is linked to the Shannon entropy and the relative α-entropy is linked with the Renyi entropy when the prior mismatched probability is uniform over the finite space. To derive such a relation for our general relative (α,β)-entropy, let us assume μ(Ω)<∞ and let *U* denote the uniform probability measure on Ω. Then, we get

(17) $$\begin{array}{c} {\mathrm{RE}}_{\alpha ,\beta }\left(P,U\right)=\frac{1}{\beta }\left[\log\mu \left(\Omega \right)-E_{\alpha ,\beta }\left(P\right)\right],\;\;\;\beta \neq 0 \end{array}$$

where the functional $E_{\alpha ,\beta }\left(P\right)$ is given in Definition 2 below and coincides with the Renyi entropy at β=1. Thus, it can be used to define a two-parameter generalization of the Renyi entropy as follows.

**Definition** **2** (Generalized Renyi   Entropy)**.** *For any probability measure P over a measurable space *Ω*, we define the generalized Renyi entropy (GRE) of order (α,β) as*

(18) $$\begin{array}{ccc} E_{\alpha ,\beta }\left(P\right) & = & \frac{1}{\beta -\alpha }\log\left[\frac{{\left(\int p^{\alpha }d\mu \right)}^{\beta }}{{\left(\int p^{\beta }d\mu \right)}^{\alpha }}\right],\;\;\;\alpha >0,\beta \in R,\;\beta \neq 0,\alpha ; \end{array}$$

(19) $$\begin{array}{ccc} E_{\alpha ,\alpha }\left(P\right) & = & -\frac{\int \log\left(p\right)p^{\alpha }d\mu }{\int p^{\alpha }d\mu }+\frac{1}{\alpha }\log\left(\int p^{\alpha }d\mu \right),\;\;\;\alpha >0. \end{array}$$

*Note that, at β=1, we have $E_{\alpha ,1}\left(P\right)=E_{\alpha }\left(P\right)$, the usual Renyi entropy measure of order α.*

The GRE is a new entropy to the best of our knowledge, and does not belong to the general class of entropy functionals as given in [104] which covers many existing entropies (including most, if not all, classical entropies). The following property of the functional $E_{\alpha ,\beta }\left(P\right)$ is easy to verify and justifies its use as a new entropy functional. To keep the focus of the present paper clear on the relative (α,β)-entropy, further properties of the GRE will be explored in our future work.

**Theorem** **1** (Entropic characteristics of GRE)**.** *For any probability measure P over a finite measure space *Ω*, we have $0\leq E_{\alpha ,\beta }\left(P\right)\leq \log\mu \left(\Omega \right)$ for all α>0 and β∈R∖{0}. The two extremes are attained as follows.*

*1*  $E_{\alpha ,\beta }\left(P\right)=0$ if P is degenerate at a point in *Ω* (no uncertainty).
*2*  $E_{\alpha ,\beta }\left(P\right)=\log\mu \left(\Omega \right)$ *if P is uniform over *Ω* (maximum uncertainty).*

**Example** **1** (Normal Distribution)**.** *Consider distributions $P_{i}$ from the most common class of multivariate (s-dimensional) normal distributions having mean $\mu_{i}\in R^{s}$ and variance matrix $\Sigma_{i}$ for i=1,2. It is known that the Shannon and the Renyi entropies of $P_{1}$ are, respectively, given by*

$$\begin{array}{ccc} E(P_{1}) & = & \frac{s}{2}+\frac{s}{2}\log\left(2\pi \right)+\frac{1}{2}\log\left|\Sigma_{1}\right|,\\ E_{\alpha }\left(P_{1}\right) & = & \frac{s}{2}\frac{\log\alpha }{\alpha -1}+\frac{s}{2}\log\left(2\pi \right)+\frac{1}{2}\log\left|\Sigma_{1}\right|,\;\;\alpha >0,\alpha \neq 1. \end{array}$$

*With the new entropy measure, GRE, the entropy of the normal distribution $P_{1}$ can be seen to have the form*

$$\begin{array}{ccc} E_{\alpha ,\beta }\left(P_{1}\right) & = & \frac{s}{2}\frac{\left(\alpha \log\beta -\beta \log\alpha \right)}{\left(\beta -\alpha \right)}+\frac{s}{2}\log\left(2\pi \right)+\frac{1}{2}\log|\Sigma_{1}|,\;\;\alpha >0,\beta \in R\setminus \left\{0,\alpha \right\},\\ E_{\alpha ,\alpha }\left(P_{1}\right) & = & \frac{s}{2}\left(1-\log\alpha \right)+\frac{s}{2}\log\left(2\pi \right)+\frac{1}{2}\log\left|\Sigma_{1}\right|,\;\;\alpha >0. \end{array}$$

*Interestingly, the GRE of a normal distribution is effectively the same as its Shannon entropy or Renyi entropy up to an additive constant. However, similar characteristic does not hold between the relative entropy (KLD) and relative (α,β)-entropy. The KLD measure between two normal distributions $P_{1}$ and $P_{2}$ is given by*

$$\begin{array}{ccc} \mathrm{RE}(P_{1},P_{2}) & = & \frac{1}{2}Trace\left(\Sigma_{2}^{-1}\Sigma_{1}\right)+\frac{1}{2}{\left(\mu_{2}-\mu_{1}\right)}^{T}\Sigma_{2}^{-1}\left(\mu_{2}-\mu_{1}\right)+\frac{1}{2}\log\left(\frac{|\Sigma_{2}|}{|\Sigma_{1}|}\right)-\frac{s}{2}, \end{array}$$

*whereas the general relative (α,β)-entropy, with α>0 and β∈R∖{0,α}, has the form*

$$\begin{array}{ccc} {\mathrm{RE}}_{\alpha ,\beta }\left(P_{1},P_{2}\right) & = & \frac{\alpha }{2}{\left(\mu_{2}-\mu_{1}\right)}^{T}{\left[\beta \Sigma_{2}+\left(\alpha -\beta \right)\Sigma_{1}\right]}^{-1}\left(\mu_{2}-\mu_{1}\right)\\ & & \;\;\;\;+\frac{1}{2\beta (\beta -\alpha )}\log\left(\frac{|\Sigma_{2}{|}^{\beta }{\left|\Sigma_{1}\right|}^{\alpha -\beta }}{|\beta \Sigma_{2}+\left(\alpha -\beta \right)\Sigma_{1}{|}^{\alpha }}\right)-\frac{s\alpha \log\alpha }{2\beta (\alpha -\beta )}. \end{array}$$

*Note that the relative (α,β)-entropy gives a more general divergence measure which utilizes different weights for the variance (or precision) matrix of the two normal distributions.*

**Example** **2** (Exponential Distribution)**.** *Consider the exponential distribution P having density $p_{\theta }\left(x\right)=\theta e^{-\theta x}I\left(x\geq 0\right)$ with θ>0. This distribution is very useful in lifetime modeling and reliability engineering; it is also the maximum entropy distribution of a non-negative random variable with fixed mean. The Shannon and the Renyi entropies of P are, respectively, given by*

$$\begin{array}{c} E\left(P\right)=1-\log\theta ,\;\;\;and\;\;E_{\alpha }\left(P\right)=\frac{\log\alpha }{\alpha -1}-\log\theta ,\;\;\alpha >0,\alpha \neq 1. \end{array}$$

*A simple calculation leads to the following form of the our new GRE measure of the exponential distribution P.*

$$\begin{array}{ccc} E_{\alpha ,\beta }\left(P\right) & = & \frac{\left(\alpha \log\beta -\beta \log\alpha \right)}{\left(\beta -\alpha \right)}-\log\theta ,\;\;\alpha >0,\beta \in R\setminus \left\{0,\alpha \right\},\\ E_{\alpha ,\alpha }\left(P\right) & = & (1-\log\alpha )-\log\theta ,\;\;\alpha >0. \end{array}$$

*Once again, the new GRE is effectively the same as the Shannon entropy or the Renyi entropy, up to an additive constant, for the exponential distribution as well.*

*Further, if $P_{1}$ and $P_{2}$ are two exponential distributions with parameters $\theta_{1}$ and $\theta_{2}$, respectively, the relative entropy (KLD) and the relative (α,β)-entropy between them are given by*

$$\begin{array}{ccc} \mathrm{RE}(P_{1},P_{2}) & = & \frac{\theta_{2}}{\theta_{1}}+\log\theta_{1}-\log\theta_{2}-1,\\ {\mathrm{RE}}_{\alpha ,\beta }\left(P_{1},P_{2}\right) & = & \frac{\alpha }{\beta (\alpha -\beta )}\log\left[\beta \theta_{1}+\left(\alpha -\beta \right)\theta_{2}\right]-\frac{1}{\alpha -\beta }\log\theta_{1}-\frac{1}{\beta }\log\theta_{2}-\frac{\alpha \log\alpha }{\beta (\alpha -\beta )}, \end{array}$$

*for α>0 and β∈R∖{0,α}. Clearly, the contributions of both the distribution is weighted differently by β and (α−β) in their relative (α,β)-entropy measure.*

Before concluding this section, we study the nature of our relative (α,β)-entropy as α→0. For this purpose, we restrict ourselves to the case of finite measure spaces with μ(Ω)<∞. It is again straightforward to note that $\lim_{\alpha \to 0}{\mathrm{RE}}_{\alpha ,\beta }\left(P,Q\right)=0$ for any β∈R and any distributions *P* and *Q* on Ω. However, if we take the limit after scaling the relative entropy measure by α we get a non-degenerate divergence measure as follows.

$$\begin{array}{ccc} {\mathrm{RE}}_{\beta }^{*}\left(P,Q\right) & = & \lim_{\alpha ↓0}\frac{1}{\alpha }{\mathrm{RE}}_{\alpha ,\beta }\left(P,Q\right)=\frac{1}{\beta^{2}}\left[\log\int {\left(\frac{p}{q}\right)}^{\beta }d\mu -\frac{\beta }{\mu (\Omega )}\int \log\left(\frac{p}{q}\right)d\mu -\log\mu \left(\Omega \right)\right], \end{array}$$

for β∈R∖{0}, and

$$\begin{array}{ccc} {\mathrm{RE}}_{0}^{*}\left(P,Q\right) & = & \lim_{\alpha ↓0}\frac{1}{\alpha }{\mathrm{RE}}_{\alpha ,0}\left(P,Q\right)=\frac{1}{2\mu (\Omega )}\left[\int {\left\{\log\left(p/q\right)\right\}}^{2}d\mu -\frac{1}{\mu (\Omega )}{\left\{\int \log\left(p/q\right)d\mu \right\}}^{2}\right]. \end{array}$$

These interesting relative entropy measures again define a subfamily of valid statistical divergences, from its construction. The particular member at β=1 is linked to the LDPD (or the γ-divergence) with tuning parameter −1 and can be thought of as a logarithmic extension of the famous Itakura–Saito divergence [105] given by

(20) $$\begin{array}{c} D_{IS}\left(P,Q\right)=\int \left(\frac{p}{q}\right)d\mu -\int \log\left(\frac{p}{q}\right)d\mu -\mu \left(\Omega \right). \end{array}$$

This Itakura–Saito-divergence has been successfully applied to non-negative matrix factorization in different applications [106] which can be extended by using the new divergence family ${\mathrm{RE}}_{\beta }^{*}\left(P,Q\right)$ in future works.

## 3. Geometry of the Relative (α,β)-Entropy

### 3.1. Continuity

We start the exploration of the geometric properties of the relative (α,β)-entropy with its continuity over the functional space $L_{\alpha }\left(\mu \right)$. In the following, we interchangeably use the notation ${\mathrm{RE}}_{\alpha ,\beta }\left(p,q\right)$ and $D_{\lambda }\left(p,q\right)$ to denote ${\mathrm{RE}}_{\alpha ,\beta }\left(P,Q\right)$ and $D_{\lambda }\left(P,Q\right)$, respectively. Our results generalize the corresponding properties of the relative α-entropy from [16,73] to our relative (α,β)-entropy or equivalent LSD measure.

**Proposition** **5.** For a given $q\in L_{\alpha }\left(\mu \right)$, consider the function $p\mapsto {\mathrm{RE}}_{\alpha ,\beta }\left(p,q\right)$ from $p\in L_{\alpha }\left(\mu \right)$ to [0,∞]. This function is lower semi-continuous in $L_{\alpha }\left(\mu \right)$ for any α>0, β∈R. Additionally, it is continuous in $L_{\alpha }\left(\mu \right)$ when α>β>0 and the relative entropy is finitely defined.

**Proof.**  First let us consider any α>0 and take $p_{n}\to p$ in $L_{\alpha }\left(\mu \right)$. Then, $\left|\right|p_{n}{\left|\right|}_{\alpha }\to {\left||p|\right|}_{\alpha }$. Also, $|p_{n}^{\alpha }-p^{\alpha }\left|\leq \right|p_{n}{|}^{\alpha }+{\left|p\right|}^{\alpha }$ and hence a general version of the dominated convergence theorem yields $p_{n}^{\alpha }\to p^{\alpha }$ in $L_{1}\left(\mu \right)$. Thus, we get

(21) $$\begin{array}{c} p_{n,\alpha }:=\frac{p_{n}^{\alpha }}{\int p_{n}^{\alpha }d\mu }\to p_{\alpha }\;\;\;\mathrm{in}\;L_{1}\left(\mu \right). \end{array}$$

Further, following ([107], Lemma 1), we know that the function $h\to \int \phi_{\lambda }\left(h\right)d\nu$ is lower semi-continuous in $L_{1}\left(\nu \right)$ for any λ∈R and any probability measure ν on (Ω,A). Taking $\nu =Q_{\alpha }$, we get from (21) that $p_{n,\alpha }/q_{\alpha }\to p_{\alpha }/q_{\alpha }$ in $L_{1}\left(\nu \right)$. Therefore, the above lower semi-continuity result along with (9) implies that

(22) $$\begin{array}{c} \underset{n\to \infty }{lim inf}D_{\lambda }\left(p_{n,\alpha },q_{\alpha }\right)\geq D_{\lambda }\left(p_{\alpha },q_{\alpha }\right)\geq 0,\;\;\lambda \in R. \end{array}$$

Now, note that the function $\psi \left(u\right)=\frac{1}{\rho }\log\left(sign\left(\rho \right)u+1\right)$ is continuous and increasing on [0,∞) for ρ>0 and on [0,1) for ρ<0. Thus, combining (22) with the definition of the relative (α,β)-entropy in (13), we get that

(23) $$\begin{array}{c} \underset{n\to \infty }{lim inf}{\mathrm{RE}}_{\alpha ,\beta }\left(p_{n},q\right)\geq {\mathrm{RE}}_{\alpha ,\beta }\left(p,q\right), \end{array}$$

i.e., the function $p\mapsto {\mathrm{RE}}_{\alpha ,\beta }\left(p,q\right)$ is lower semi-continuous.

Finally, consider the case α>β>0. Note that the dual space of $L_{\alpha /\beta }\left(\mu \right)$ is $L_{\frac{\alpha }{\alpha -\beta }}\left(\mu \right)$ since α>β>0. Also, for $q\in L_{\alpha }\left(\mu \right)$, we have ${\left(\frac{q}{{\left||q|\right|}_{\alpha }}\right)}^{\alpha -\beta }\in L_{\frac{\alpha }{\alpha -\beta }}\left(\mu \right)$, the dual space of the Banach space $L_{\alpha /\beta }\left(\mu \right)$. Therefore, the function $T:L_{\alpha /\beta }\left(\mu \right)\mapsto R$ defined by

$$T\left(h\right)=\int h{\left(\frac{q}{{\left||q|\right|}_{\alpha }}\right)}^{\alpha -\beta }d\mu ,\;\;h\in L_{\alpha /\beta }\left(\mu \right),$$

is a bounded linear functional and hence continuous. Now, take $p_{n}\to p$ in $L_{\alpha }\left(\mu \right)$ so that $\left|\right|p_{n}{\left|\right|}_{\alpha }\to {\left||p|\right|}_{\alpha }$ as n→∞. Therefore, $\left(\frac{p_{n}}{\left|\right|p_{n}{\left|\right|}_{\alpha }}\right)\to \left(\frac{p}{{\left||p|\right|}_{\alpha }}\right)$ in $L_{\alpha }\left(\mu \right)$ implying ${\left(\frac{p_{n}}{\left|\right|p_{n}{\left|\right|}_{\alpha }}\right)}^{\beta }\to {\left(\frac{p}{{\left||p|\right|}_{\alpha }}\right)}^{\beta }$ in $L_{\alpha /\beta }\left(\mu \right)$. Hence, by the continuity of *T* on $L_{\alpha /\beta }\left(\mu \right)$, we get

$$T\left({\left(\frac{p_{n}}{\left|\right|p_{n}{\left|\right|}_{\alpha }}\right)}^{\beta }\right)\to T\left({\left(\frac{p}{{\left||p|\right|}_{\alpha }}\right)}^{\beta }\right),\;\;\mathrm{as}\;\;n\to \infty .$$

However, from (14), we get

(24) $$\begin{array}{c} {\mathrm{RE}}_{\alpha ,\beta }\left(p_{n},q\right)=\frac{\alpha }{\beta (\beta -\alpha )}\log T\left({\left(\frac{p_{n}}{\left|\right|p_{n}{\left|\right|}_{\alpha }}\right)}^{\beta }\right)\to \frac{\alpha }{\beta (\beta -\alpha )}\log T\left({\left(\frac{p}{{\left||p|\right|}_{\alpha }}\right)}^{\beta }\right)={\mathrm{RE}}_{\alpha ,\beta }\left(p,q\right). \end{array}$$

This proves the continuity of ${\mathrm{RE}}_{\alpha ,\beta }\left(p,q\right)$ in its first argument when α>β>0. ☐

**Remark** **2.** Whenever *Ω* is finite (discrete) equipped with the counting measure μ, all integrals in the definition of ${\mathrm{RE}}_{\alpha ,\beta }\left(P,Q\right)$ become finite sums and any limit can be taken inside these finite sums. Thus, whenever defined finitely, the function $p\mapsto {\mathrm{RE}}_{\alpha ,\beta }\left(p,q\right)$ is always continuous in this case.

**Remark** **3.** For a general infinite space *Ω*, the function $p\mapsto {\mathrm{RE}}_{\alpha ,\beta }\left(p,q\right)$ is not necessarily continuous for the cases α<β. This can be seen by using the same counterexample as given in Remark 3 of [16]. However, it is yet to be verified if this function can be continuous for β<0 cases.

**Proposition** **6.** For a given $p\in L_{\alpha }\left(\mu \right)$, consider the function $q\mapsto {\mathrm{RE}}_{\alpha ,\beta }\left(p,q\right)$ from $q\in L_{\alpha }\left(\mu \right)$ to [0,∞]. This function is lower semi-continuous in $L_{\alpha }\left(\mu \right)$ for any α>0 and β∈R.

**Proof.**  Fix an α>0 and β∈R, which in turn fixes a λ∈R. Note that, the relative (α,β)-entropy measure can be re-expressed from (13) as

(25) $$\begin{array}{c} {\mathrm{RE}}_{\alpha ,\beta }\left(p,q\right)=\frac{1}{\beta \lambda }\log\left[sign\left(\beta \lambda \right)D_{-(\lambda +1)}\left(q_{\alpha },p_{\alpha }\right)+1\right]. \end{array}$$

Now, consider a sequence $q_{n}\to q$ in $L_{\alpha }\left(\mu \right)$ and proceed as in the proof of Proposition 5 using ([107], Lemma 1) to obtain

(26) $$\begin{array}{c} \underset{n\to \infty }{lim inf}D_{-(\lambda +1)}\left(q_{n,\alpha },p_{\alpha }\right)\geq D_{-(\lambda +1)}\left(q_{\alpha },p_{\alpha }\right)\geq 0,\;\;\lambda \in R. \end{array}$$

Now, whenever $D_{-(\lambda +1)}\left(q_{\alpha },p_{\alpha }\right)=1$ with βλ<0 or $D_{-(\lambda +1)}\left(q_{\alpha },p_{\alpha }\right)=\infty$ with βλ>0, we get from (25) and (26) that

(27) $$\begin{array}{c} \underset{n\to \infty }{lim inf}{\mathrm{RE}}_{\alpha ,\beta }\left(p,q_{n}\right)={\mathrm{RE}}_{\alpha ,\beta }\left(p,q\right)=+\infty . \end{array}$$

In all other cases, we consider the function $\psi \left(u\right)=\frac{1}{\rho }\log\left(sign\left(\rho \right)u+1\right)$ as in the proof of Proposition 5. This function is continuous and increasing whenever the corresponding relative entropy is finitely defined for all tuning parameter values; on [0,∞) for ρ>0 and on [0,1) for ρ<0. Hence, again combining (26) with (25) through the function ψ, we conclude that

(28) $$\begin{array}{c} \underset{n\to \infty }{lim inf}{\mathrm{RE}}_{\alpha ,\beta }\left(p,q_{n}\right)\geq {\mathrm{RE}}_{\alpha ,\beta }\left(p,q\right). \end{array}$$

Therefore, the function $q\mapsto {\mathrm{RE}}_{\alpha ,\beta }\left(p,q\right)$ is also lower semi-continuous. ☐

**Remark** **4.** As in Remark 2, whenever *Ω* is finite (discrete) and is equipped with the counting measure μ, the function $q\mapsto {\mathrm{RE}}_{\alpha ,\beta }\left(p,q\right)$ is continuous in $L_{\alpha }\left(\mu \right)$ for any fixed $p\in L_{\alpha }\left(\mu \right)$, α>0 and β∈R.

### 3.2. Convexity

It has been shown in [16] that the relative α-entropy (i.e., ${\mathrm{RE}}_{\alpha ,1}\left(p,q\right)$) is neither convex nor bi-convex, but it is quasi-convex in *p*. For general β≠1, however, the relative (α,β)-entropy ${\mathrm{RE}}_{\alpha ,\beta }\left(p,q\right)$ is not even quasi-convex in $p\in L_{\alpha }\left(\mu \right)$; rather it is quasi-convex on the β-power transformed space of densities, $L_{\alpha }{\left(\mu \right)}^{\beta }=\left\{p^{\beta }:p\in L_{\alpha }\left(\mu \right)\right\}$, as described in the following theorem. Note that, for α,β>0, $L_{\alpha }{\left(\mu \right)}^{\beta }=L_{\alpha /\beta }\left(\mu \right)$. Here we define the lower level set $B_{\alpha ,\beta }\left(q,r\right)=\left\{p:{\mathrm{RE}}_{\alpha ,\beta }\left(p,q\right)\leq r\right\}$ and its power-transformed set $B_{\alpha ,\beta }{\left(q,r\right)}^{\beta }=\left\{p^{\beta }:p\in B_{\alpha ,\beta }\left(q,r\right)\right\}$, for any $q\in L_{\alpha }\left(\mu \right)$ and r>0.

**Theorem** **2.** For any given α>0, β∈R and $q\in L_{\alpha }\left(\mu \right)$, the sets $B_{\alpha ,\beta }{\left(q,r\right)}^{\beta }$ are convex for all r>0. Therefore, the function $p^{\beta }\mapsto {\mathrm{RE}}_{\alpha ,\beta }\left(p,q\right)$ is quasi-convex on $L_{\alpha }{\left(\mu \right)}^{\beta }$.

**Proof.**  Note that, at β=1, our theorem coincides with Proposition 5 of [16]; so we will prove the result for the case β≠1. Fix α,r>0, a real β∉{1,α}, $q\in L_{\alpha }\left(\mu \right)$, and $p_{0},p_{1}\in B_{\alpha ,\beta }\left(q,r\right)$. Then $p_{0}^{\beta },p_{1}^{\beta }\in B_{\alpha ,\beta }{\left(q,r\right)}^{\beta }$. For τ∈[0,1], we consider $p_{\tau }^{\beta }=\tau p_{1}^{\beta }+\bar{\tau }p_{0}^{\beta }$ with $\bar{\tau }=1-\tau$. We need to show that $p_{\tau }^{\beta }\in B_{\alpha ,\beta }{\left(q,r\right)}^{\beta }$, i.e., ${\mathrm{RE}}_{\alpha ,\beta }\left(p_{\tau },q\right)\leq r$.

Now, from (14), we have

(29) $$\begin{array}{c} {\mathrm{RE}}_{\alpha ,\beta }\left(p,q\right)=\frac{1}{\beta \lambda }\log\int {\left(\frac{p}{{\left||p|\right|}_{\alpha }}\right)}^{\beta }{\left(\frac{q}{{\left||q|\right|}_{\alpha }}\right)}^{\alpha -\beta }d\mu =\frac{1}{\beta \lambda }\log\int {\left(\frac{p_{\alpha }}{q_{\alpha }}\right)}^{\beta /\alpha }dQ_{\alpha }. \end{array}$$

Since $p_{0}^{\beta },p_{1}^{\beta }\in B_{\alpha ,\beta }{\left(q,r\right)}^{\beta }$, we have

(30) $$\begin{array}{c} sign\left(\beta \lambda \right)\int {\left(\frac{p_{\tau }}{\left|\right|p_{\tau }{\left|\right|}_{\alpha }}\right)}^{\beta }{\left(\frac{q}{{\left||q|\right|}_{\alpha }}\right)}^{\alpha -\beta }d\mu \leq sign\left(\beta \lambda \right)e^{r\beta \lambda },\;\;\;\;\mathrm{for}\;\tau =0,1. \end{array}$$

For any τ∈(0,1), we get

(31) $$\begin{array}{cccc} sign\left(\beta \lambda \right)\int {\left(\frac{p_{\tau }}{\left|\right|p_{\tau }{\left|\right|}_{\alpha }}\right)}^{\beta }{\left(\frac{q}{{\left||q|\right|}_{\alpha }}\right)}^{\alpha -\beta }d\mu & = & sign\left(\beta \lambda \right)\int \left(\frac{\tau p_{1}^{\beta }+\bar{\tau }p_{0}^{\beta }}{\left|\right|p_{\tau }{\left|\right|}_{\alpha }^{\beta }}\right){\left(\frac{q}{{\left||q|\right|}_{\alpha }}\right)}^{\alpha -\beta }d\mu , & \;\;\;\;\;\;\;\;\;\left[\mathrm{by}\;\mathrm{definition}\;\mathrm{of}\;p_{\tau }\right]\\ & \leq & sign\left(\beta \lambda \right)e^{r\beta \lambda }\frac{\tau ||p_{1}{\left|\right|}_{\alpha }^{\beta }+\bar{\tau }\left|\right|p_{0}{\left|\right|}_{\alpha }^{\beta }}{\left|\right|p_{\tau }{\left|\right|}_{\alpha }^{\beta }},\;\;\;\;\;\;\left[\mathrm{by}\;(30)\right]. \end{array}$$

Now, using the extended Minkowski’s inequalities from Lemma 1, given below, along with (31) and noting that βλ=β(β−α)/α, we get that

$$\begin{array}{ccc} sign\left(\beta \lambda \right)\int {\left(\frac{p_{\tau }}{\left|\right|p_{\tau }{\left|\right|}_{\alpha }}\right)}^{\beta }{\left(\frac{q}{{\left||q|\right|}_{\alpha }}\right)}^{\alpha -\beta }d\mu & \leq & sign\left(\beta \lambda \right)e^{r\beta \lambda }. \end{array}$$

Therefore, by (29) and the fact that $\frac{1}{\rho }\log\left(sign\left(\rho \right)u\right)$ is increasing in *u*, we finally get ${\mathrm{RE}}_{\alpha ,\beta }\left(p_{\tau },q\right)\leq r$. This proves the result for α≠β.

The case β=α can be proved in a similar manner and is left as an exercise to the readers. ☐

**Lemma** **1** (Extended Minkowski’s inequality)**.** *Fix α>0, a real β∉{1,α}, $p_{0},p_{1}\in L_{\alpha }\left(\mu \right)$, and τ∈[0,1]. Define $p_{\tau }^{\beta }=\tau p_{1}^{\beta }+\bar{\tau }p_{0}^{\beta }$ with $\bar{\tau }=1-\tau$. Then we have the following inequalities:*

(32) $$\begin{array}{ccc} \left|\right|p_{\tau }{\left|\right|}_{\alpha }^{\beta } & \geq & \tau ||p_{1}{\left|\right|}_{\alpha }^{\beta }+\bar{\tau }\left|\right|p_{0}{\left|\right|}_{\alpha }^{\beta },\;\;\;if\;\beta \left(\beta -\alpha \right)>0, \end{array}$$

(33) $$\begin{array}{ccc} \left|\right|p_{\tau }{\left|\right|}_{\alpha }^{\beta } & \leq & \tau ||p_{1}{\left|\right|}_{\alpha }^{\beta }+\bar{\tau }\left|\right|p_{0}{\left|\right|}_{\alpha }^{\beta },\;\;\;if\;\beta \left(\beta -\alpha \right)<0. \end{array}$$

**Proof.**  It follows by using the Jensen’s inequality and the convexity of the function $x^{\beta /\alpha }$. ☐

Next, note in view of Proposition 4 that, for any $p,q\in L_{\alpha }\left(\mu \right)$, ${\mathrm{RE}}_{\alpha ,\beta }\left(p,q\right)={\mathrm{RE}}_{\alpha ,\alpha -\beta }\left(q,p\right)$. Using this result along with the above theorem, we also get the quasi-convexity of the relative (α,β)-entropy ${\mathrm{RE}}_{\alpha ,\beta }\left(p,q\right)$ in *q* over a different power transformed space of densities. This leads to the following theorem.

**Theorem** **3.** For any given α>0, β∈R and $p\in L_{\alpha }\left(\mu \right)$, the function $q^{\alpha -\beta }\mapsto {\mathrm{RE}}_{\alpha ,\beta }\left(p,q\right)$ is quasi-convex on $L_{\alpha }{\left(\mu \right)}^{\alpha -\beta }$. In particular, for the choice β=α−1, the function $q\mapsto {\mathrm{RE}}_{\alpha ,\beta }\left(p,q\right)$ is quasi-convex on $L_{\alpha }\left(\mu \right)$.

**Remark** **5.** Note that, at α=β=1, the ${\mathrm{RE}}_{1,1}\left(p,q\right)$ coincides with the KLD measure (or relative entropy) which is quasi-convex in both the arguments p and q on $L_{\alpha }\left(\mu \right)$.

### 3.3. Extended Pythagorean Relation

Motivated by the quasi-convexity of ${\mathrm{RE}}_{\alpha ,\beta }\left(p,q\right)$ on $L_{\alpha }{\left(\mu \right)}^{\beta }$, we now present a Pythagorean-type result for the general relative (α,β)-entropy over the power-transformed space. It generalizes the corresponding result for relative α-entropy [16]; the proof is similar to that in [16] with necessary modifications due to the transformation of the domain space.

**Theorem** **4** (Pythagorean Property)**.** *Fix an α>0, β∈R with β≠α and $p_{0},p_{1},q\in L_{\alpha }\left(\mu \right)$. Define $p_{\tau }\in L_{\alpha }\left(\mu \right)$ by $p_{\tau }^{\beta }=\tau p_{1}^{\beta }+\bar{\tau }p_{0}^{\beta }$ for τ∈[0,1] and $\bar{\tau }=1-\tau$.*

*(i)*  *Suppose ${\mathrm{RE}}_{\alpha ,\beta }\left(p_{0},q\right)$ and ${\mathrm{RE}}_{\alpha ,\beta }\left(p_{1},q\right)$ are finite. Then, ${\mathrm{RE}}_{\alpha ,\beta }\left(p_{\tau },q\right)\geq {\mathrm{RE}}_{\alpha ,\beta }\left(p_{0},q\right)$ for all τ∈[0,1], i.e., the back-transformation of line segment joining $p_{1}^{\beta }$ and $p_{0}^{\beta }$ on $L_{\alpha }{\left(\mu \right)}^{\beta }$ to $L_{\alpha }\left(\mu \right)$ does not intersect $B_{\alpha ,\beta }\left(q,{\mathrm{RE}}_{\alpha ,\beta }\left(p_{0},q\right)\right)$, if and only if*
  (34) $$\begin{array}{c} {\mathrm{RE}}_{\alpha ,\beta }\left(p_{1},q\right)\geq {\mathrm{RE}}_{\alpha ,\beta }\left(p_{1},p_{0}\right)+{\mathrm{RE}}_{\alpha ,\beta }\left(p_{0},q\right). \end{array}$$
*(ii)*  *Suppose ${\mathrm{RE}}_{\alpha ,\beta }\left(p_{\tau },q\right)$ is finite for some fixed τ∈(0,1). Then, the back-transformation of line segment joining $p_{1}^{\beta }$ and $p_{0}^{\beta }$ on $L_{\alpha }{\left(\mu \right)}^{\beta }$ to $L_{\alpha }\left(\mu \right)$ does not intersect $B_{\alpha ,\beta }\left(q,{\mathrm{RE}}_{\alpha ,\beta }\left(p_{\tau },q\right)\right)$ if and only if*
  (35) $$\begin{array}{ccc} {\mathrm{RE}}_{\alpha ,\beta }\left(p_{1},q\right) & = & {\mathrm{RE}}_{\alpha ,\beta }\left(p_{1},p_{\tau }\right)+{\mathrm{RE}}_{\alpha ,\beta }\left(p_{\tau },q\right), \end{array}$$
  (36) $$\begin{array}{ccc} and\;\;{\mathrm{RE}}_{\alpha ,\beta }\left(p_{0},q\right) & = & {\mathrm{RE}}_{\alpha ,\beta }\left(p_{0},p_{\tau }\right)+{\mathrm{RE}}_{\alpha ,\beta }\left(p_{\tau },q\right). \end{array}$$

**Proof** **of Part (i).** Let $P_{\tau ,\alpha }$ to be the probability measure having μ-density $p_{\tau ,\alpha }=\frac{p_{\tau }^{\alpha }}{\int p_{\tau }^{\alpha }d\mu }$ for τ∈[0,1]. Also note that, with λ=β/α−1, we have

(37) $$\begin{array}{c} D_{\lambda }\left(P_{\alpha },Q_{\alpha }\right)=sign\left(\beta \lambda \right)\left[\int {\left(\frac{p}{{\left||p|\right|}_{\alpha }}\right)}^{\beta }{\left(q_{\alpha }\right)}^{-\lambda }d\mu -1\right],\;\;\mathrm{for}p,q\in L_{\alpha }\left(\mu \right). \end{array}$$

Thus, (34) is equivalent to the statement

(38) $$\begin{array}{c} sign\left(\beta \lambda \right)||p_{0}{\left|\right|}_{\alpha }^{\beta }\int p_{1}^{\beta }{\left(q_{\alpha }\right)}^{-\lambda }d\mu \geq sign\left(\beta \lambda \right)\int p_{1}^{\beta }{\left(p_{0,\alpha }\right)}^{-\lambda }d\mu \cdot \int p_{0}^{\beta }{\left(q_{\alpha }\right)}^{-\lambda }d\mu . \end{array}$$

and we have

(39) $$\begin{array}{c} D_{\lambda }\left(P_{\tau ,\alpha },Q_{\alpha }\right)=sign\left(\beta \lambda \right)\left[\int {\left(\frac{p_{\tau }}{\left|\right|p_{\tau }{\left|\right|}_{\alpha }}\right)}^{\beta }{\left(q_{\alpha }\right)}^{-\lambda }d\mu -1\right]=sign\left(\beta \lambda \right)\frac{s(\tau )}{t(\tau )}, \end{array}$$

where $s\left(\tau \right)=\int p_{\tau }^{\beta }{\left(q_{\alpha }\right)}^{-\lambda }d\mu$ and $t\left(\tau \right)=\left|\right|p_{\tau }{\left|\right|}_{\alpha }^{\beta }$. Now consider the two implications separately.

*Only if statement:* Now, let us assume that ${\mathrm{RE}}_{\alpha ,\beta }\left(p_{\tau },q\right)\geq {\mathrm{RE}}_{\alpha ,\beta }\left(p_{0},q\right)$ for all τ∈(0,1). Then, we get $\frac{1}{\tau }\left[D_{\lambda }\left(P_{\tau ,\alpha },Q_{\alpha }\right)-D_{\lambda }\left(P_{0,\alpha },Q_{\alpha }\right)\right]\geq 0$ for all τ∈(0,1). Letting τ↓0, we get that

(40) $$\begin{array}{c} {\left.\frac{\partial }{\partial \tau }D_{\lambda }\left(P_{\tau ,\alpha },Q_{\alpha }\right)\right|}_{\tau =0}\geq 0. \end{array}$$

In order to find the derivative of $D_{\lambda }\left(P_{\tau ,\alpha },Q_{\alpha }\right)$, we first note that

$$\begin{array}{ccc} \frac{s(\tau )-s(0)}{\tau } & = & \frac{1}{\tau }\left[\int p_{\tau }^{\beta }{\left(q_{\alpha }\right)}^{-\lambda }d\mu -\int p_{0}^{\beta }{\left(q_{\alpha }\right)}^{-\lambda }d\mu \right]=\int \left(p_{1}^{\beta }-p_{0}^{\beta }\right){\left(q_{\alpha }\right)}^{-\lambda }d\mu , \end{array}$$

and hence

(41) $$\begin{array}{ccc} s^{\prime }\left(0\right) & = & \lim_{\tau ↓0}\frac{s(\tau )-s(0)}{\tau }=\int \left(p_{1}^{\beta }-p_{0}^{\beta }\right){\left(q_{\alpha }\right)}^{-\lambda }d\mu . \end{array}$$

Further, using a simple modification of the techniques in the proof of ([16], Theorem 9), it is easy to verify that the derivative of t(τ) with respect to τ exists and is given by

$$t^{\prime }\left(\tau \right)={\left(\int p_{\tau }^{\alpha }d\mu \right)}^{\frac{\left(\beta -\alpha \right)}{\alpha }}\int p_{\tau }^{\alpha -\beta }\left(p_{1}^{\beta }-p_{0}^{\beta }\right)d\mu .$$

Hence we get

(42) $$\begin{array}{ccc} t^{\prime }\left(0\right) & = & {\left(\int p_{0}^{\alpha }d\mu \right)}^{\frac{\left(\beta -\alpha \right)}{\alpha }}\int p_{0}^{\alpha -\beta }\left(p_{1}^{\beta }-p_{0}^{\beta }\right)d\mu =\int p_{1}^{\beta }{\left(p_{0,\alpha }\right)}^{-\lambda }d\mu -\left|\right|p_{0}{\left|\right|}_{\alpha }^{\beta }. \end{array}$$

Therefore, the derivative of $D_{\lambda }\left(P_{\tau ,\alpha },Q_{\alpha }\right)=sign\left(\beta \lambda \right)s\left(\tau \right)/t\left(\tau \right)$ exists and is given by $sign\left(\beta \lambda \right)\left[t\left(0\right)s^{\prime }\left(0\right)-t^{\prime }\left(0\right)s\left(0\right)\right]/t{\left(0\right)}^{2}$. Therefore, using (40), we get that

(43) $$\begin{array}{c} sign\left(\beta \lambda \right)t\left(0\right)s^{\prime }\left(0\right)\geq sign\left(\beta \lambda \right)t^{\prime }\left(0\right)s\left(0\right), \end{array}$$

which implies (38) after substituting the values from (41) and (42).

*If statement:* Now, let us assume that (34)—or equivalently (38)—holds true. Further, as in the derivation of (38), we can start from the trivial statement

$${\mathrm{RE}}_{\alpha ,\beta }\left(p_{0},q\right)={\mathrm{RE}}_{\alpha ,\beta }\left(p_{0},p_{0}\right)+{\mathrm{RE}}_{\alpha ,\beta }\left(p_{0},q\right),$$

to deduce

(44) $$\begin{array}{c} sign\left(\beta \lambda \right)||p_{0}{\left|\right|}_{\alpha }^{\beta }\int p_{0}^{\beta }{\left(q_{\alpha }\right)}^{-\lambda }d\mu =sign\left(\beta \lambda \right)\int p_{0}^{\beta }{\left(p_{0,\alpha }\right)}^{-\lambda }d\mu \cdot \int p_{0}^{\beta }{\left(q_{\alpha }\right)}^{-\lambda }d\mu . \end{array}$$

Now, multiply (38) by τ and (44) by $\bar{\tau }$, and add to get

$$\begin{array}{c} sign\left(\beta \lambda \right)||p_{0}{\left|\right|}_{\alpha }^{\beta }\int p_{\tau }^{\beta }{\left(q_{\alpha }\right)}^{-\lambda }d\mu \geq sign\left(\beta \lambda \right)\int p_{\tau }^{\beta }{\left(p_{0,\alpha }\right)}^{-\lambda }d\mu \cdot \int p_{0}^{\beta }{\left(q_{\alpha }\right)}^{-\lambda }d\mu . \end{array}$$

In view of (37), this implies that

$$\begin{array}{c} {\mathrm{RE}}_{\alpha ,\beta }\left(p_{\tau },q\right)\geq {\mathrm{RE}}_{\alpha ,\beta }\left(p_{\tau },p_{0}\right)+{\mathrm{RE}}_{\alpha ,\beta }\left(p_{0},q\right)\geq {\mathrm{RE}}_{\alpha ,\beta }\left(p_{0},q\right). \end{array}$$

This proves the if statement of Part (i) completing the proof. ☐

**Proof** **of Part (ii).** Note that the *if statement* follows directly from Part (i).

To prove the *only if statement*, we first show that ${\mathrm{RE}}_{\alpha ,\beta }\left(p_{1},q\right)$ and ${\mathrm{RE}}_{\alpha ,\beta }\left(p_{0},q\right)$ are finite since ${\mathrm{RE}}_{\alpha ,\beta }\left(p_{\tau },q\right)$ is finite. For this purpose, we note that $p_{1}^{\beta }\leq \tau^{-1}p_{\tau }^{\beta }$ by the definition of $p_{\tau }$ and hence ${\left(p_{1}/q\right)}^{\beta }\leq \tau^{-1}{\left(p_{\tau }/q\right)}^{\beta }$. Therefore, we get

(45) $$\begin{array}{c} {\left(\frac{p_{1,\alpha }}{q_{\alpha }}\right)}^{\beta /\alpha }={\left(\frac{p_{1}}{q}\right)}^{\beta }{\left(\frac{\left||q|\right|}{\left|\right|p_{1}\left|\right|}\right)}^{\beta }\leq \frac{1}{\tau }{\left(\frac{p_{\tau }}{q}\right)}^{\beta }{\left(\frac{\left||q|\right|}{\left|\right|p_{1}\left|\right|}\right)}^{\beta }=\frac{1}{\tau }{\left(\frac{p_{\tau ,\alpha }}{q_{\alpha }}\right)}^{\beta }{\left(\frac{\left|\right|p_{\tau }\left|\right|}{\left|\right|p_{1}\left|\right|}\right)}^{\beta }. \end{array}$$

Integration with respect to $Q_{\alpha }$ and using (29), we get ${\mathrm{RE}}_{\alpha ,\beta }\left(p_{1},q\right)\leq {\mathrm{RE}}_{\alpha ,\beta }\left(p_{\tau },q\right)+c<\infty ,$ where *c* is a constant. Similarly one can also show that ${\mathrm{RE}}_{\alpha ,\beta }\left(p_{0},q\right)<\infty$.

Therefore, we can apply Part (i) to conclude that

(46) $$\begin{array}{c} {\mathrm{RE}}_{\alpha ,\beta }\left(p_{1},q\right)\geq {\mathrm{RE}}_{\alpha ,\beta }\left(p_{1},p_{\tau }\right)+{\mathrm{RE}}_{\alpha ,\beta }\left(p_{\tau },q\right),\;\mathrm{and}\;{\mathrm{RE}}_{\alpha ,\beta }\left(p_{0},q\right)\geq {\mathrm{RE}}_{\alpha ,\beta }\left(p_{0},p_{\tau }\right)+{\mathrm{RE}}_{\alpha ,\beta }\left(p_{\tau },q\right). \end{array}$$

These relations imply that

(47) $$\begin{array}{c} sign\left(\beta \lambda \right)||p_{\tau }{\left|\right|}_{\alpha }^{\beta }\int p_{1}^{\beta }{\left(q_{\alpha }\right)}^{-\lambda }d\mu \geq sign\left(\beta \lambda \right)\int p_{1}^{\beta }{\left(p_{\tau ,\alpha }\right)}^{-\lambda }d\mu \cdot \int p_{\tau }^{\beta }{\left(q_{\alpha }\right)}^{-\lambda }d\mu , \end{array}$$

(48) $$\begin{array}{c} \mathrm{and}\;sign\left(\beta \lambda \right)||p_{\tau }{\left|\right|}_{\alpha }^{\beta }\int p_{0}^{\beta }{\left(q_{\alpha }\right)}^{-\lambda }d\mu \geq sign\left(\beta \lambda \right)\int p_{0}^{\beta }{\left(p_{\tau ,\alpha }\right)}^{-\lambda }d\mu \cdot \int p_{\tau }^{\beta }{\left(q_{\alpha }\right)}^{-\lambda }d\mu . \end{array}$$

The proof of the above results proceed in a manner analogous to the proof of (38). Now, if either of the inequalities in (46) is strict, the corresponding inequality in (47) or (48) will also be strict. Then, multiplying (47) and (48) by τ and $\bar{\tau }$, respectively, and adding them we get (44) with a strict inequality (in place of an equality), which is a contradiction. Hence, both inequalities in (46) must be equalities implying (35) and (36). This completes the proof. ☐

Note that, at β=1, the above theorem coincides with Theorem 9 of [16]. However, for general α,β as well, the above extended Pythagorean relation for the relative (α,β)-entropy suggests that it behaves “like" a squared distance (although with a non-linear space transformation). So, one can meaningfully define its projection on to a suitable set which we will explore in the following sections.

## 4. The Forward Projection of Relative (α,β)-Entropy

The forward projection, i.e., minimization with respect to the first argument given a fixed second argument, leads to the important maximum entropy principle of information theory; it also relates to the Gibbs conditioning principle from statistical physics [16]. Let us now formally define and study the forward projection of the relative (α,β)-entropy. Let $S^{*}$ denote the set of probability measure on (Ω,A) and let the set of corresponding μ-densities be denoted by $S=\left\{p=dP/d\mu :P\in S^{*}\right\}$.

**Definition** **3** (Forward (α,β)-Projection)**.** *Fix $Q\in S^{*}$ having μ-density $q\in L_{\alpha }\left(\mu \right)$. Let E⊂S with ${\mathrm{RE}}_{\alpha ,\beta }\left(p,q\right)<\infty$ for some p∈E. Then, $p^{*}\in E$ is called the forward projection of the relative (α,β)-entropy or simply the forward (α,β)-projection (or forward LSD projection) of q on E if it satisfies the relation*

(49) $$\begin{array}{c} {\mathrm{RE}}_{\alpha ,\beta }\left(p^{*},q\right)=\underset{p\in E}{\inf}{\mathrm{RE}}_{\alpha ,\beta }\left(p,q\right). \end{array}$$

Note that we must assume that, $E\subset L_{\alpha }\left(\mu \right)$ so that the above relative (α,β)-entropy is finitely defined for p∈E.

We first prove the uniqueness of the forward (α,β)-projection from the Pythagorean property, whenever it exists. The following theorem describe the connection of the forward (α,β)-projection with Pythagorean relation; the proof is same as that of ([16], Theorem 10) using Theorem 4 and hence omitted for brevity.

**Theorem** **5.** *Consider the set E⊂S such that $E^{\beta }$ is convex and fix $q\in L_{\alpha }\left(\mu \right)$. Then, $p^{*}\in E\cap B_{\alpha ,\beta }\left(q,\infty \right)$ is a forward (α,β)-projection of q on E if and only if every $p\in E\cap B_{\alpha ,\beta }\left(q,\infty \right)$ satisfies*

(50) $$\begin{array}{c} {\mathrm{RE}}_{\alpha ,\beta }\left(p,q\right)\geq {\mathrm{RE}}_{\alpha ,\beta }\left(p,p^{*}\right)+{\mathrm{RE}}_{\alpha ,\beta }\left(p^{*},q\right). \end{array}$$

*Further, if ${\left(p^{*}\right)}^{\beta }$ is an algebraic inner point of $E^{\beta }$, i.e., for every p∈E there exists $p^{\prime }\in E$ and τ∈(0,1) such that ${\left(p^{*}\right)}^{\beta }=\tau p^{\beta }+\left(1-\tau \right){\left(p^{\prime }\right)}^{\beta }$, then every p∈E satisfies ${\mathrm{RE}}_{\alpha ,\beta }\left(p,q\right)<\infty$ and*

$$\begin{array}{c} {\mathrm{RE}}_{\alpha ,\beta }\left(p,q\right)={\mathrm{RE}}_{\alpha ,\beta }\left(p,p^{*}\right)+{\mathrm{RE}}_{\alpha ,\beta }\left(p^{*},q\right),\;\;and\;\;{\mathrm{RE}}_{\alpha ,\beta }\left(p^{\prime },q\right)={\mathrm{RE}}_{\alpha ,\beta }\left(p^{\prime },p^{*}\right)+{\mathrm{RE}}_{\alpha ,\beta }\left(p^{*},q\right). \end{array}$$

**Corollary** **1** (Uniqueness of Forward (α,β)-Projection)**.** Consider the set E⊂S such that $E^{\beta }$ is convex and fix $q\in L_{\alpha }\left(\mu \right)$. If a forward (α,β)-projection of q on E exists, it must be unique a.s.[μ].

**Proof.**  Suppose $p_{1}^{*}$ and $p_{2}^{*}$ are two forward (α,β)-projection of *q* on E. Then, by definition, ${\mathrm{RE}}_{\alpha ,\beta }\left(p_{1}^{*},q\right)={\mathrm{RE}}_{\alpha ,\beta }\left(p_{2}^{*},q\right)<\infty$. Applying Theorem 5 with $p^{*}=p_{1}^{*}$ and $p=p_{2}^{*}$, we get

$$\begin{array}{c} {\mathrm{RE}}_{\alpha ,\beta }\left(p_{2}^{*},q\right)\geq {\mathrm{RE}}_{\alpha ,\beta }\left(p_{2}^{*},p_{1}^{*}\right)+{\mathrm{RE}}_{\alpha ,\beta }\left(p_{1}^{*},q\right). \end{array}$$

Hence ${\mathrm{RE}}_{\alpha ,\beta }\left(p_{2}^{*},p_{1}^{*}\right)\leq 0$ or ${\mathrm{RE}}_{\alpha ,\beta }\left(p_{2}^{*},p_{1}^{*}\right)=0$ by non-negativity of relative entropy, which further implies that $p_{1}^{*}=p_{2}^{*}$ a.s.[μ] by Proposition 1. ☐

Next we will show the existence of the forward (α,β)-projection under suitable conditions. We need to use an extended Apollonius Theorem for the ϕ-divergence measure $D_{\lambda }$ used in the definition (13) of the relative (α,β)-entropy. Such a result is proved in [16] for the special case α(1+λ)=1; the following lemma extends it for the general case α(1+λ)=β∈R.

**Lemma** **2.** *Fix $p_{0},p_{1},q\in L_{\alpha }\left(\mu \right)$, τ∈[0,1] and α(1+λ)=β∈R with α>0 and define r satisfying*

(51) $$\begin{array}{c} r^{\beta }=\frac{\frac{\tau }{\left|\right|p_{1}{\left|\right|}_{\alpha }^{\beta }}p_{1}^{\beta }+\frac{1-\tau }{\left|\right|p_{0}{\left|\right|}_{\alpha }^{\beta }}p_{0}^{\beta }}{\frac{\tau }{\left|\right|p_{1}{\left|\right|}_{\alpha }^{\beta }}+\frac{1-\tau }{\left|\right|p_{0}{\left|\right|}_{\alpha }^{\beta }}}. \end{array}$$

*Let $p_{j,\alpha }=p_{j}^{\alpha }/\int p_{j}^{\alpha }d\mu$ for j=0,1, and similarly $q_{\alpha }$ and $r_{\alpha }$. Then, if β(β−α)>0 we have*

(52) $$\begin{array}{c} \tau D_{\lambda }\left(p_{1,\alpha },q_{\alpha }\right)+\left(1-\tau \right)D_{\lambda }\left(p_{0,\alpha },q_{\alpha }\right)\geq \tau D_{\lambda }\left(p_{1,\alpha },r_{\alpha }\right)+\left(1-\tau \right)D_{\lambda }\left(p_{0,\alpha },r_{\alpha }\right)+D_{\lambda }\left(r_{\alpha },q_{\alpha }\right), \end{array}$$

*but the inequality gets reversed if β(β−α)<0.*

**Proof.**  By (37), we get

$$\begin{array}{c} \tau D_{\lambda }\left(p_{1,\alpha },q_{\alpha }\right)+\left(1-\tau \right)D_{\lambda }\left(p_{0,\alpha },q_{\alpha }\right)-\tau D_{\lambda }\left(p_{1,\alpha },r_{\alpha }\right)-\left(1-\tau \right)D_{\lambda }\left(p_{0,\alpha },r_{\alpha }\right)\\ =sign\left(\beta \lambda \right)\tau \int {\left(\frac{p_{1}}{\left|\right|p_{1}{\left|\right|}_{\alpha }}\right)}^{\beta }\left[{\left(q_{\alpha }\right)}^{-\lambda }-{\left(r_{\alpha }\right)}^{-\lambda }\right]d\mu +sign\left(\beta \lambda \right)\left(1-\tau \right)\int {\left(\frac{p_{0}}{\left|\right|p_{0}{\left|\right|}_{\alpha }}\right)}^{\beta }\left[{\left(q_{\alpha }\right)}^{-\lambda }-{\left(r_{\alpha }\right)}^{-\lambda }\right]d\mu \\ ={sign\left(\beta \lambda \right)||r||}_{\alpha }^{\beta }\left[\frac{\tau }{\left|\right|p_{1}{\left|\right|}_{\alpha }^{\beta }}+\frac{1-\tau }{\left|\right|p_{0}{\left|\right|}_{\alpha }^{\beta }}\right]\int {\left(\frac{r}{{\left||r|\right|}_{\alpha }}\right)}^{\beta }\left[{\left(q_{\alpha }\right)}^{-\lambda }-{\left(r_{\alpha }\right)}^{-\lambda }\right]d\mu \\ ={sign\left(\beta \lambda \right)||r||}_{\alpha }^{\beta }\left[\frac{\tau }{\left|\right|p_{1}{\left|\right|}_{\alpha }^{\beta }}+\frac{1-\tau }{\left|\right|p_{0}{\left|\right|}_{\alpha }^{\beta }}\right]D_{\lambda }\left(R_{\alpha },Q_{\alpha }\right). \end{array}$$

Then the Lemma follows by an application of the extended Minkowski’s inequalities (32) and (33) from Lemma 1. ☐

We now present the sufficient conditions for the existence of the forward (α,β)-projection in the following theorem.

**Theorem 6** (Existence of Forward (α,β)-Projection)**.** Fix α>0 and β∈R with β≠α and $q\in L_{\alpha }\left(\mu \right)$. Given any set E⊂S for which $E^{\beta }$ is convex and closed and ${\mathrm{RE}}_{\alpha ,\beta }\left(p,q\right)<\infty$ for some p∈E, a forward (α,β)-projection of q on E always exists (and it is unique by Corollary 1).

**Proof.**  We prove it separately for the cases βλ>0 and βλ<0, extending the arguments from [16]. The case βλ=0 can be obtained from these two cases by standard limiting arguments and hence omitted for brevity.

The Case βλ>0:

Consider a sequence $\{p_{n}\}\subset E$ such that $D_{\lambda }\left(p_{n,\alpha },q_{\alpha }\right)<\infty$ for each *n* and $D_{\lambda }\left(p_{n,\alpha },q_{\alpha }\right)\to \underset{p\in E}{\inf}D_{\lambda }\left(p_{\alpha },q_{\alpha }\right)$ as n→∞. Then, by Lemma 2 applied to $p_{m}$ and $p_{n}$ with τ=1/2, we get

(53) $$\begin{array}{c} \frac{1}{2}D_{\lambda }\left(p_{m,\alpha },q_{\alpha }\right)+\frac{1}{2}D_{\lambda }\left(p_{n,\alpha },q_{\alpha }\right)\geq \frac{1}{2}D_{\lambda }\left(p_{m,\alpha },r_{m,n,\alpha }\right)+\frac{1}{2}D_{\lambda }\left(p_{n,\alpha },r_{m,n,\alpha }\right)+D_{\lambda }\left(r_{m,n,\alpha },q_{\alpha }\right), \end{array}$$

where $r_{m,n}$ is defined by

(54) $$\begin{array}{c} r_{m,n}^{\beta }=\frac{\frac{\tau }{\left|\right|p_{m}{\left|\right|}_{\alpha }^{\beta }}p_{m}^{\beta }+\frac{1-\tau }{\left|\right|p_{n}{\left|\right|}_{\alpha }^{\beta }}p_{n}^{\beta }}{\frac{\tau }{\left|\right|p_{m}{\left|\right|}_{\alpha }^{\beta }}+\frac{1-\tau }{\left|\right|p_{n}{\left|\right|}_{\alpha }^{\beta }}}. \end{array}$$

Note that, since $E^{\beta }$ is convex, $r_{m,n}\in E^{\beta }$ and so $r_{m,n}\in E$. Also, using the non-negativity of divergence, (53) leads to

(55) $$\begin{array}{c} 0\leq \frac{1}{2}D_{\lambda }\left(p_{m,\alpha },r_{m,n,\alpha }\right)+\frac{1}{2}D_{\lambda }\left(p_{n,\alpha },r_{m,n,\alpha }\right)\leq \frac{1}{2}D_{\lambda }\left(p_{m,\alpha },q_{\alpha }\right)+\frac{1}{2}D_{\lambda }\left(p_{n,\alpha },q_{\alpha }\right)-D_{\lambda }\left(r_{m,n,\alpha },q_{\alpha }\right). \end{array}$$

Taking limit as m,n→∞, one can see that $\left[\frac{1}{2}D_{\lambda }\left(p_{m,\alpha },q_{\alpha }\right)+\frac{1}{2}D_{\lambda }\left(p_{n,\alpha },q_{\alpha }\right)-D_{\lambda }\left(r_{m,n,\alpha },q_{\alpha }\right)\right]\to 0$ and hence $\left[D_{\lambda }\left(p_{m,\alpha },r_{m,n,\alpha }\right)+D_{\lambda }\left(p_{n,\alpha },r_{m,n,\alpha }\right)\right]\to 0$. Thus, $D_{\lambda }\left(p_{m,\alpha },r_{m,n,\alpha }\right)\to 0$ as m,n→∞ by non-negativity. This along with a generalization of Pinker’s inequality for ϕ-divergence ([100], Theorem 1) gives

(56) $$\begin{array}{c} \lim_{m,n\to \infty }\left|\right|p_{m,\alpha }-r_{m,n,\alpha }{\left|\right|}_{T}=0, \end{array}$$

whenever λ(1+λ)>0 (which is true since βλ>0); here $\left|\right|\cdot {\left|\right|}_{T}$ denotes the total variation norm. Now, by triangle inequality

$$\left|\right|p_{m,\alpha }-p_{n,\alpha }{\left|\right|}_{T}\leq \left|\right|p_{m,\alpha }-r_{m,n,\alpha }{\left|\right|}_{T}+\left|\right|p_{n,\alpha }-r_{m,n,\alpha }{\left|\right|}_{T}\to 0,\;\;\mathrm{as}\;\;m,n\to \infty .$$

Thus, $\left\{p_{n,\alpha }\right\}$ is Cauchy in $L_{1}\left(\mu \right)$ and hence converges to some $g\in L_{1}\left(\mu \right)$, i.e.,

(57) $$\begin{array}{c} \lim_{n\to \infty }\int \left|p_{n,\alpha }-g\right|d\mu =0, \end{array}$$

and *g* is a probability density with respect to μ since each $p_{n}$ is so. Also, (57) implies that $p_{n,\alpha }\to g$ in [μ]-measure and hence $p_{n,\alpha }^{1/\alpha }\to g^{1/\alpha }$ in $L_{\alpha }\left(\mu \right)$ by an application of generalized dominated convergence theorem.

Next, as in the proof of ([16], Theorem 8), we can show that $\left|\right|p_{n}{\left|\right|}_{\alpha }$ is bounded and hence $\left|\right|p_{n}{\left|\right|}_{\alpha }\to c$ for some c>0, possibly working with a subsequence if needed. Thus we have $p_{n}=\left|\right|p_{n}{\left|\right|}_{\alpha }p_{n,\alpha }^{1/\alpha }\to cg^{1/\alpha }$ in $L_{\alpha }\left(\mu \right)$. However, since $E^{\beta }$ is closed, we have E is closed and hence $cg^{1/\alpha }=p^{*}$ for some $p^{*}\in E$. Further, since ∫gdμ=1, we must have $c=\left|\right|p^{*}{\left|\right|}_{\alpha }$ and hence $g=p_{\alpha }^{*}$. Since $p_{n}\to p^{*}$ and $p^{*}\in E$, Proposition 5 implies that

$${\mathrm{RE}}_{\alpha ,\beta }\left(p^{*},q\right)\leq \underset{n\to \infty }{lim inf}{\mathrm{RE}}_{\alpha ,\beta }\left(p_{n},q\right)=\underset{p\in E}{\inf}{\mathrm{RE}}_{\alpha ,\beta }\left(p,q\right)\leq {\mathrm{RE}}_{\alpha ,\beta }\left(p^{*},q\right),$$

where the second equality follows by continuity of the function $f\left(u\right)={\left(\beta \lambda \right)}^{-1}\log\left(sign\left(\beta \lambda \right)u+1\right)$, definitions of $p_{n}$ sequence and (13). Hence, we must have ${\mathrm{RE}}_{\alpha ,\beta }\left(p^{*},q\right)=\underset{p\in E}{\inf}{\mathrm{RE}}_{\alpha ,\beta }\left(p,q\right)$, i.e., $p^{*}$ is a forward (α,β)-projection of *q* on E.

The Case βλ<0:

Note that, in this case, we must have 0<β<α, since α>0. Then, using (29), we can see that

(58) $$\begin{array}{ccc} \underset{p\in E}{\inf}{\mathrm{RE}}_{\alpha ,\beta }\left(p,q\right) & = & \frac{1}{\beta \lambda }\log\left[\underset{p\in E}{\sup}\int {\left(\frac{p}{{\left||p|\right|}_{\alpha }}\right)}^{\beta }{\left(\frac{q}{{\left||q|\right|}_{\alpha }}\right)}^{\alpha -\beta }d\mu \right]\\ & = & \frac{1}{\beta \lambda }\log\left[\underset{h\in \tilde{E}}{\sup}\int hgd\mu \right], \end{array}$$

where $g={\left(\frac{q}{{\left||q|\right|}_{\alpha }}\right)}^{\alpha -\beta }\in L_{\frac{\alpha }{\alpha -\beta }}\left(\mu \right)$ and

$$\tilde{E}=\left\{s{\left(\frac{p}{{\left||p|\right|}_{\alpha }}\right)}^{\beta }:p\in E,s\in \left[0,1\right]\right\}\subset L_{\alpha /\beta }\left(\mu \right).$$

Now, since $E^{\beta }$ and hence E is closed, one can show that $\tilde{E}$ is also closed; see, e.g., the proof of ([16], Theorem 8). Next, we will show that $\tilde{E}$ is also convex. For take $s_{1}{\left(\frac{p_{1}}{\left|\right|p_{1}{\left|\right|}_{\alpha }}\right)}^{\beta }\in \tilde{E}$ and $s_{0}{\left(\frac{p_{0}}{\left|\right|p_{0}{\left|\right|}_{\alpha }}\right)}^{\beta }\in \tilde{E}$ for some $s_{0},s_{1}\in \left[0,1\right]$ and $p_{0},p_{1}\in E$, and take any τ∈[0,1]. Note that

$$\tau s_{1}{\left(\frac{p_{1}}{\left|\right|p_{1}{\left|\right|}_{\alpha }}\right)}^{\beta }+\left(1-\tau \right)s_{0}{\left(\frac{p_{0}}{\left|\right|p_{0}{\left|\right|}_{\alpha }}\right)}^{\beta }=s_{\tau }{\left(\frac{p_{\tau }}{\left|\right|p_{\tau }{\left|\right|}_{\alpha }}\right)}^{\beta },$$

where

$$p_{\tau }^{\beta }=\frac{\tau s_{1}{\left(\frac{p_{1}}{\left|\right|p_{1}{\left|\right|}_{\alpha }}\right)}^{\beta }+\left(1-\tau \right)s_{0}{\left(\frac{p_{0}}{\left|\right|p_{0}{\left|\right|}_{\alpha }}\right)}^{\beta }}{\frac{\tau s_{1}}{\left|\right|p_{1}{\left|\right|}_{\alpha }^{\beta }}+\frac{\left(1-\tau \right)s_{0}}{\left|\right|p_{0}{\left|\right|}_{\alpha }^{\beta }}},\;\;\mathrm{and}\;\;s_{\tau }=\left[\frac{\tau s_{1}}{\left|\right|p_{1}{\left|\right|}_{\alpha }^{\beta }}+\frac{\left(1-\tau \right)s_{0}}{\left|\right|p_{0}{\left|\right|}_{\alpha }^{\beta }}\right]\left|\right|p_{\tau }{\left|\right|}_{\alpha }^{\beta }.$$

However, by convexity of $E^{\beta }$, $p_{\tau }\in E$ and also $0\leq s_{\tau }\leq 1$ by the extended Minkowski inequality (33). Therefore, $s_{\tau }{\left(\frac{p_{\tau }}{\left|\right|p_{\tau }{\left|\right|}_{\alpha }}\right)}^{\beta }\in \tilde{E}$ and hence $\tilde{E}$ is convex.

Finally, since 0<β<α, $L_{\alpha /\beta }\left(\mu \right)$ is a reflexive Banach space and hence the closed and convex $\tilde{E}\subset L_{\alpha /\beta }\left(\mu \right)$ is also closed in the weak topology. So, the unit ball is compact in the weak topology by the Banach-Alaoglu theorem and hence its closed subset $\tilde{E}$ is also weakly compact. However, since *g* belongs to the dual space of $L_{\alpha /\beta }\left(\mu \right)$, the linear functional h↦∫hgdμ is continuous in weak topology and also increasing in *s*. Hence its supremum over $\tilde{E}$ is attained at s=1 and some $p^{*}\in E$, which is the required forward (α,β)-projection. ☐

Before concluding this section, we will present one example of the forward (α,β)-projection onto a transformed-linear family of distributions.

**Example 3** (An example of the forward (α,β)-projection)**.** *Fix α>0, β∈R∖{0,α} and $q\in L_{\alpha }\left(\mu \right)$ related to the measure Q. Consider measurable functions $f_{i}:\Omega \mapsto R$ for i∈I, an index set, and the family of distributions*

$$L_{\beta }^{*}=\left\{P\in S^{*}:\int f_{\gamma }dP_{\beta }=0\right\}\subset S^{*}.$$

*Let us denote the corresponding μ-density set by $L_{\beta }=\left\{p=\frac{dP}{d\mu }:P\in L_{\beta }^{*}\right\}.$ We assume that, $L_{\beta }^{*}$ is non-empty, every $P\in L_{\beta }^{*}$ is absolute continuous with respect to μ and $L_{\beta }\subset L_{\alpha }\left(\mu \right)$.*

*Then, $p^{*}$ is the forward (α,β)-projection of q on $L_{\beta }$ if and only if there exists a function g in the $L_{1}\left(Q_{\beta }\right)$-closure of the linear space spanned by $\left\{f_{i}:i\in I\right\}$ and a subset N⊂Ω such that, for every $P\in L_{\beta }^{*}$*

$$\begin{array}{c} \left\{\begin{array}{cc} P(N)=0 & if\;\alpha <\beta ,\\ c\int_{N}q^{\alpha -\beta }dP_{\beta }\leq \int_{\Omega \setminus N}gdP_{\beta } & if\;\alpha >\beta , \end{array}\right. \end{array}$$

*with $c=\frac{\int {\left(p^{*}\right)}^{\alpha }d\mu }{\int {\left(p^{*}\right)}^{\beta }q^{\alpha -\beta }d\mu }$ and $p^{*}$ satisfies*

$$\begin{array}{c} p^{*}{\left(x\right)}^{\alpha -\beta }=cq{\left(x\right)}^{\alpha -\beta }+g\left(x\right),\;\;\;\;if\;x\notin N,\\ p^{*}\left(x\right)=0,\;\;\;\;\;\;\;\;\;\;\;\;\;\;\;if\;x\in N. \end{array}$$

*The proof follows by extending the arguments of the proof of ([16], Theorem 11) and hence it is left as an exercise to the readers.*

**Remark** **6.** Note that, at the special case β=1, $L_{1}^{*}$ is a linear family of distributions and the above example coincides with ([16], Theorem 11) on the forward projection of relative α-entropy on $L_{1}^{*}$. However, it is still an open question to derive the forward (α,β)-projection on $L_{1}^{*}$.

## 5. Statistical Applications: The Minimum Relative Entropy Inference

### 5.1. The Reverse Projection and Parametric Estimation

As in the case of the forward projection of a relative entropy measure, we can also define the reverse projection by minimizing it with respect to the second argument over a convex set E keeping the first argument fixed. More formally, we use the following definition.

**Definition** **4** (Reverse (α,β)-Projection)**.** *Fix $p\in L_{\alpha }\left(\mu \right)$ and let E⊂S with ${\mathrm{RE}}_{\alpha ,\beta }\left(p,q\right)<\infty$ for some q∈E. Then, $q^{*}\in E$ is called the reverse projection of the relative (α,β)-entropy or simply the reverse (α,β)-projection (or reverse LSD projection) of p on E if it satisfies the relation*

(59) $$\begin{array}{c} {\mathrm{RE}}_{\alpha ,\beta }\left(p,q^{*}\right)=\underset{q\in E}{\inf}{\mathrm{RE}}_{\alpha ,\beta }\left(p,q\right). \end{array}$$

We can get sufficient conditions for the existence and uniqueness of the reverse (α,β)-projection directly from Theorem 6 and the fact that ${\mathrm{RE}}_{\alpha ,\beta }\left(p,q\right)={\mathrm{RE}}_{\alpha ,\alpha -\beta }\left(q,p\right)$; this is presented in the following theorem.

**Theorem 7** (Existence and Uniqueness of Reverse (α,β)-Projection)**.** Fix α>0 and β∈R with β≠α and $p\in L_{\alpha }\left(\mu \right)$. Given any set E⊂S for which $E^{\alpha -\beta }$ is convex and closed and ${\mathrm{RE}}_{\alpha ,\beta }\left(p,q\right)<\infty$ for some q∈E, a reverse (α,β)-projection of p on E exists and is unique.

The reverse projection is mostly used in statistical inference where we fix the first argument of a relative entropy measure (or divergence measure) at the empirical data distribution and minimize the relative entropy with respect to the model family of distributions in its second argument. The resulting estimator, commonly known as the minimum distance or minimum divergence estimator, yields the reverse projection of the observed data distribution on the family of model distributions with respect to the relative entropy or divergence under consideration. This approach was initially studied by [9,10,11,12,13] to obtain the popular maximum likelihood estimator as the reverse projection with respect to the relative entropy in (2). More recently, this approach has become widely popular, but with more general relative entropies or divergence measures, to obtain robust estimators against possible contamination in the observed data. Let us describe it more rigorously in the following for our relative (α,β)-entropy.

Suppose we have independent and identically distributed data $X_{1},\ldots ,X_{n}$ from a true distribution *G* having density *g* with respect to some common dominating measure μ. We model *g* by a parametric model family of μ-densities $F=\{f_{\theta }:\theta \in \Theta \subseteq R^{p}\}$, where it is assumed that both *g* and $f_{\theta }$ have the same support independent of θ. Our objective is to infer about the unknown parameter θ. In minimum divergence inference, an estimator of θ is obtained by minimizing the divergence measure between (an estimate of) *g* and $f_{\theta }$ with respect to θ∈Θ. Maji et al. [78] have considered the LSD (or equivalently the relative (α,β)-entropy) as the divergence under consideration and defined the corresponding minimum divergence functional at *G*, say $T_{\alpha ,\beta }\left(G\right)$, through the relation

(60) $${\mathrm{RE}}_{\alpha ,\beta }\left(g,f_{T_{\alpha ,\beta }\left(G\right)}\right)=\min_{\theta \in \Theta }{\mathrm{RE}}_{\alpha ,\beta }\left(g,f_{\theta }\right),$$

whenever the minimum exists. We will refer to $T_{\alpha ,\beta }\left(G\right)$ as the minimum relative (α,β)-entropy (MRE) functional, or the minimum LSD functional in the language of [78,79]. Note that, if g∈F, i.e., $g=f_{\theta_{0}}$ for some $\theta_{0}\in \Theta$, then we must have $T_{\alpha ,\beta }\left(G\right)=\theta_{0}$. If g∉F, we call $T_{\alpha ,\beta }\left(G\right)$ as the “best fitting parameter" value, since $f_{T_{\alpha ,\beta }\left(G\right)}$ is the closest model element to *g* in the LSD sense. In fact, for g∉F, $T_{\alpha ,\beta }\left(G\right)$ is nothing but the reverse (α,β)-projection of the true density *g* on the model family F, which exists and is unique under the sufficient conditions of Theorem 7. Therefore, under identifiability of the model family F we get the existence and uniqueness of the MRE functional, which is presented in the following corollary. Although this estimator was first introduced by [78] in terms of the LSD, the results concerning the existence of the estimate were not provided.

**Corollary** **2** (Existence and Uniqueness of the MRE Functional)**.** *Consider the above parametric estimation problem with $g\in L_{\alpha }\left(\mu \right)$ and $F\subset L_{\alpha }\left(\mu \right)$. Fix α>0 and β∈R with β≠α and assume that the model family F is identifiable in **θ**.*

*(1)*  Suppose $g=f_{\theta_{0}}$ for some $\theta_{0}\in \Theta$. Then the unique MRE functional is given by $T_{\alpha ,\beta }\left(G\right)=\theta_{0}$.
*(2)*  Suppose g∉F. If $F^{\alpha -\beta }$ is convex and closed and ${\mathrm{RE}}_{\alpha ,\beta }\left(g,f_{\theta }\right)<\infty$ for some θ∈Θ, the MRE functional $T_{\alpha ,\beta }\left(G\right)$ exists and is unique.

Further, under standard differentiability assumptions, we can obtain the estimating equation of the MRE functional $T_{\alpha ,\beta }\left(G\right)$ as given by

(61) $$\left[\int f_{\theta }^{\alpha }u_{\theta }d\mu \right]\left[\int f_{\theta }^{\alpha -\beta }g^{\beta }d\mu \right]=\left[\int f_{\theta }^{\alpha -\beta }g^{\beta }u_{\theta }d\mu \right]\left[\int f_{\theta }^{\alpha }d\mu \right],$$

where $u_{\theta }\left(x\right)=\frac{\partial }{\partial \theta }\ln f_{\theta }\left(x\right)$. It is important to note that, at β=α=1, the MRE functional $T_{1,1}\left(G\right)$ coincides with the maximum likelihood functional since ${\mathrm{RE}}_{1,1}=\mathrm{RE}$, the KLD measure. Based on the estimating Equation (61), Maji et al. [78] extensively studied the theoretical robustness properties of the MRE functional against gross-error contamination in data through the higher order influence function analysis. The classical first order influence function was seen to be inadequate for this purpose; it becomes independent of β at the model but the real-life performance of the MRE functional critically depends on both α and β [78,79] as we will also see in Section 5.2.

In practice, however, the true data generating density is not known and so we need to use some empirical estimate in place of *g* and the resulting value of the MRE functional is called the minimum relative (α,β)-entropy estimator (MREE) or the minimum LSD estimator in the terminology of [78,79]. Note that, when the data are discrete and μ is the counting measure, one can use a simple estimate of *g* given by the relative frequencies $r_{n}\left(x\right)=\frac{1}{n}\sum_{i=1}^{n}I\left(X_{i}=x\right)$, where I(A) is the indicator function of the event *A*; the corresponding MREE is then obtained by solving (61) with g(x) replaced by $r_{n}\left(x\right)$ and integrals replaced by sums over the discrete support. Asymptotic properties of this MREE under discrete models are well-studied by [78,79] for the tuning parameters α≥1 and β∈R; the same line of argument can be used to extend them also for the cases α∈(0,1) in a straightforward manner.

However, in case of continuous data, there is no such simple estimator available to use in place of *g* unless β=1. When β=1, the estimating Equation (61) depends on *g* through the terms $\int f_{\theta }^{\alpha -1}gd\mu =\int f_{\theta }^{\alpha -1}dG$ and $\int f_{\theta }^{\alpha -1}u_{\theta }gd\mu =\int f_{\theta }^{\alpha -1}u_{\theta }dG$; so we can simply use the empirical distribution function $G_{n}$ in place of *G* and solve the resulting equation to obtain the corresponding MREE. However, for β≠1, we must use a non-parametric kernel estimator $g_{n}$ of *g* in (61) to obtain the MREE under continuous models; this leads to complications including bandwidth selection while deriving the asymptotics of the resulting MREE. One possible approach to avoid such complications is to use the smoothed model technique, which has been applied in [108] for the case of minimum ϕ-divergence estimators. Another alternative approach has been discussed in [109,110]. However, the detailed analyses of the MREE under the continuous model, in either of the above approaches, are yet to be studied so far.

### 5.2. Numerical Illustration: Binomial Model

Let us now present numerical illustrations under the common binomial model to study the finite sample performance of the MREEs. Along with the known properties of the MREE at α≥1 (i.e., the minimum LSD estimators with τ≥0 from [78,79]), here we will additionally explore their properties in case of α∈(0,1) and for the new divergences ${\mathrm{RE}}_{\beta }^{*}\left(P,Q\right)$ related to α=0.

Suppose $X_{1},\ldots ,X_{n}$ are random observations from a true density *g* having support χ={0,1,2,…,m} for some positive integer *m*. We model *g* by the Binomial(m,θ) densities fθ(x)=nxθx(1−θ)m−x for x∈χ and θ∈[0,1]. Here an estimate $\hat{g}$ of *g* is given by the relative frequency $\hat{g}\left(x\right)=r_{n}\left(x\right)$. For any α>0 and β∈R, the relative (α,β)-entropy between $\hat{g}$ and $f_{\theta }$ is given by

REα,β(g^,fθ)=1βlog∑x=0mnxαθ1−θαx(1−θ)mα+1α−βlog∑x=0mrn(x)α−αβ(α−β)log∑x=0mnxα−βθ1−θ(α−β)x(1−θ)m(α−β)rn(x)β,

which can be minimized with respect to θ∈[0,1] to obtain the corresponding MREE of θ. Note that, it is also the solution of the estimating Equation (61) with g(x) replaced by the relative frequency $r_{n}\left(x\right)$. However, in this example, $u_{\theta }\left(x\right)=\frac{x-m\theta }{\theta (1-\theta )}$ and hence the MREE estimating equation simplifies to

(62) ∑x=0mnxα(x−mθ)θ1−θαx∑x=0mnxαθ1−θαx=∑x=0m(x−mθ)nxα−βθ1−θ(α−β)xrn(x)β∑x=0mnxα−βθ1−θ(α−β)xrn(x)β.

We can numerically solve the above estimating equation over θ∈[0,1], or equivalently over the transformed parameter $p:=\frac{\theta }{1-\theta }\in \left[0,\infty \right]$, to obtain the corresponding MREE (i.e., the minimum LSD estimator).

We simulate random sample of size *n* from a binomial population with true parameter $\theta_{0}=0.1$ with m=10 and numerically compute the MREE. Repeating this exercise 1000 times, we can obtain an empirical estimate of the bias and the mean squared error (MSE) of the MREE of 10θ (since θ is very small in magnitude). Table 1 and Table 2 present these values for sample sizes n=20,50,100 and different values of tuning parameters α>0 and β>0; their existences are guaranteed by Corollary 2. Note that the choice α=1=β gives the maximum likelihood estimator whereas β=1 only yields the minimum LDPD estimator with parameter α. Next, in order to study the robustness, we contaminate 10% of each sample by random observations from a distant binomial distribution with parameters θ=0.9 and m=10 and repeat the above simulation exercise; the resulting bias and MSE for the contaminated samples are given in Table 3 and Table 4. Our observations from these tables can be summarized as follows.

- Under pure data with no contamination, the maximum likelihood estimator (the MREE at α=1=β) has the least bias and MSE as expected, which further decrease as sample size increases.
- As we move away from α=1 and β=1 in either direction, the MSEs of the corresponding MREEs under pure data increase slightly; but as long as the tuning parameters remain within a reasonable window of the (1,1) point and neither component is very close to zero, this loss in efficiency is not very significant.
- When α or β approaches zero, the MREEs become somewhat unstable generating comparatively larger MSE values. This is probably due to the presence of inliers under the discrete binomial model. Note that, the relative (α,β)-entropy measures with β≤0 are not finitely defined for the binomial model if there is just only one empty cell present in the data.
- Under contamination, the bias and MSE of the maximum likelihood estimator increase significantly but many MREEs remains stable. In particular, the MREEs with β≥α and the MREEs with β close to zero are non-robust against data contamination. Many of the remaining members of the MREE family provide significantly improved robust estimators.
- In the entire simulation, the combination (α=1,β=0.7) appears to provide the most stable results. In Table 4, the best results are available along a tubular region which moves from the top left-hand to the bottom right-hand of the table subject to the conditions that α>β and none of them are very close to zero.
- Based on our numerical experiments, the optimum range of values of α, β providing the most robust minimum relative (α,β)-estimators are α=0.9,1, 0.5≤β≤0.7 and 1<α≤1.5, 0.5≤β<1. Note that this range includes the estimators based on the logarithmic power divergence measure as well as the new LSD measures with α<1.
- Many of the MREEs, which belong to the optimum range mentioned in the last item and are close to the combination α=1=β, generally also provide the best trade-off between efficiency under pure data and robustness under contaminated data.

In summary, many MREEs provide highly robust estimators under data contamination along with only a very small loss in efficiency under pure data. These numerical findings about the finite sample behavior of the MREEs under the binomial model and the corresponding optimum range of tuning parameters, for the subclass with α≥1, are consistent with the findings of [78,79] who used a Poisson model. Additionally, our illustrations shed lights on the properties of the MREEs at α<1 as well and show that some MREEs in this range, e.g., at α=0.9 and β=0.5, also yield optimum estimators in terms of the dual goal of high robustness and high efficiency.

### 5.3. Application to Testing Statistical Hypothesis

We end the paper with a very brief indication on the potential of the relative (α,β)-entropy or the LSD measure in statistical hypothesis testing problems. The minimum possible value of the relative entropy or divergence measure between the data and the null distribution indicates the amount of departure from null and hence can be used to develop a statistical testing procedure.

Consider the parametric estimation set-up as in Section 5.1 with g∈F and fix a parameter value $\theta_{0}\in \Theta$. Suppose we want to test the simple null hypothesis in the one sample case given by

$$H_{0}:\theta =\theta_{0}\;\;\;against\;\;\;\;H_{1}:\theta \neq \theta_{0}.$$

Maji et al. [78] have developed the LSD-based test statistics for the above testing problem as given by

(63) $$T_{n,\alpha ,\beta }^{\left(1\right)}=2n{\mathrm{RE}}_{\alpha ,\beta }\left(f_{{\hat{\theta }}_{\alpha ,\beta }},f_{\theta_{0}}\right),$$

where ${\hat{\theta }}_{\alpha ,\beta }$ is the MREE with parameters α and β. [78,79] have also developed the LSD-based test for a simple two-sample problem where two independent samples of sizes $n_{1}$ and $n_{2}$ are given from true densities $f_{\theta_{1}},f_{\theta_{2}}\in F$, respectively and we want to test for the homogeneity of the two samples trough the hypothesis

$$H_{0}:\theta_{1}=\theta_{2}\;\;\;against\;\;\;\;H_{1}:\theta_{1}\neq \theta_{2}.$$

The proposed test statistics for this two-sample problem has the form

(64) $$T_{n,\alpha ,\beta }^{\left(2\right)}=\frac{2n_{1}n_{2}}{n_{1}+n_{2}}{\mathrm{RE}}_{\alpha ,\beta }\left(f_{\left(1\right){\hat{\theta }}_{\alpha ,\beta }},f_{\left(2\right){\hat{\theta }}_{\alpha ,\beta }}\right),$$

where ${}^{\left(1\right)}{\hat{\theta }}_{\alpha ,\beta }$ and ${}^{\left(2\right)}{\hat{\theta }}_{\alpha ,\beta }$ are the MREEs of $\theta_{1}$ and $\theta_{2}$, respectively, obtained from the two samples separately Note that, at α=β=1, both the test statistics in (63) and (64) become asymptotically equivalent to the corresponding likelihood ratio tests under the respective null hypothesis. Maji et al. [78,79] have studied the asymptotic properties of these two tests, which have asymptotic null distributions as linear combinations of chi-square distributions. They have also numerically illustrated the benefits of these LSD or relative (α,β)-entropy-based tests, although with tuning parameters α≥1 only, to achieve robust inference against possible contamination in the sample data.

The same approach can also be used to develop robust tests for more complex hypothesis testing problems based on the relative (α,β)-entropy or the LSD measures, now with parameters α>0, and also using the new divergences ${\mathrm{RE}}_{\beta }^{*}\left(\cdot ,\cdot \right)$. For example, consider the above one sample set-up and a subset $\Theta_{0}\subset \Theta$ and let we are interested in testing the composite hypothesis

$$H_{0}:\theta \in \Theta_{0}\;\;\;against\;\;\;\;H_{1}:\theta \notin \Theta_{0}.$$

with similar motivation from (63) and (64), we can construct relative entropy or LSD-based test statistics for testing the above composite hypothesis as given by

(65) $${\tilde{T_{n,\alpha ,\beta }}}^{\left(1\right)}=2n{\mathrm{RE}}_{\alpha ,\beta }\left(f_{{\hat{\theta }}_{\alpha ,\beta }},f_{{\tilde{\theta }}_{\alpha ,\beta }}\right),$$

where ${\tilde{\theta }}_{\alpha ,\beta }$ is the restricted MREE with parameters α and β obtained by minimizing the relative entropy over $\theta \in \Theta_{0}$ and ${\hat{\theta }}_{\alpha ,\beta }$ is the corresponding unrestricted MREE obtained by minimizing over θ∈Θ. It will surely be of significant interest to study the asymptotic and robustness properties of this relative entropy-based test for the above composite hypothesis under one sample or even more general hypotheses with two or more samples. However, considering the length of the present paper, which is primarily focused on the geometric properties of entropies and relative entropies, we have deferred the detailed analyses of such MREE-based hypothesis testing procedures in a future report.

## 6. Conclusions

We have explored the geometric properties of the LSD measures through a new information theoretic formulation when we develop this divergence measure as a natural extension of the relative α-entropy; we refer to it as the two-parameter relative (α,β)-entropy. It is shown to be always lower semicontinuous in both the arguments, but is continuous in its first argument only if α>β>0. We also proved that the relative (α,β)-entropy is quasi-convex in both its arguments after a suitable (different) transformation of the domain space and derive an extended Pythagorean relation under these transformations. Along with the study of its forward and reverse projections, statistical applications are also discussed.

It is worthwhile to note that the information theoretic divergences can also be used to define new measures of robustness and efficiency of a parameter estimate; one can then obtain the optimum robust estimator, along Hampel’s infinitesimal principle, to achieve the best trade-off between these divergence-based summary measures [111,112,113]. In particular, the LDPD measure, a prominent member of our LSD or relative (α,β)-entropy family, has been used by [113] who have illustrated important theoretical properties including different types of equivariance of the resulting optimum estimators besides their strong robustness properties. A similar approach can also be used with our general relative (α,β)-entropies to develop estimators with enhanced optimality properties, establishing a better robustness-efficiency trade-off. The present work opens up several interesting problems to be solved in future research as already noted throughout the paper. In particular, we recall that the relative α-entropy has an interpretation from the problem of guessing under source uncertainty [17,71]. As an extension of relative α-entropy, a similar information theoretic interpretation of the relative (α,β)-entropy (i.e., the LSD) is expected and its proper interpretation will be a useful development. Additionally, we have obtained a new extension of the Renyi entropy as a by-product and detailed study of this new entropy measure and its potential applications may lead to a new aspect of the mathematical information theory. Also, statistical applications of these measures need to be studied thoroughly specially for the continuous models, where the complications of a kernel density estimator is unavoidable, and for testing complex composite hypotheses from one or more samples. We hope to pursue some of these interesting extensions in future.

## Figures and Tables

**Table 1:** Bias of the MREE for different α, β and sample sizes *n* under pure data.

β	α									
	0.3	0.5	0.7	0.9	1	1.1	1.3	1.5	1.7	2
n=20										
0.1	−0.210	−0.416	−0.397	−0.311	−0.277	−0.227	−0.130	0.021	0.024	0.122
0.3	2.218	−0.273	−0.229	−0.160	−0.141	−0.115	−0.096	−0.068	−0.036	0.034
0.5	−0.127	0.001	−0.125	−0.088	−0.082	−0.069	−0.058	−0.042	−0.032	−0.019
0.7	−0.093	−0.110	−0.010	−0.046	−0.044	−0.029	−0.023	−0.031	−0.023	−0.020
0.9	−0.066	−0.056	−0.028	−0.001	−0.015	−0.002	0.008	0.000	−0.006	−0.013
1	−0.041	−0.045	−0.017	0.005	−0.002	0.011	0.014	0.012	0.008	−0.003
1.3	−0.035	−0.013	0.023	0.036	0.030	0.039	0.088	0.039	0.035	0.021
1.5	−0.003	0.012	0.048	0.053	0.047	0.058	0.053	0.170	0.048	0.035
1.7	0.012	0.028	0.058	0.067	0.061	0.070	0.070	0.058	0.269	0.045
2	0.008	0.049	0.078	0.084	0.078	0.086	0.087	0.078	0.069	0.444
n=50										
0.1	−0.085	−0.301	−0.254	−0.183	−0.156	−0.106	−0.002	0.114	0.292	0.245
0.3	1.829	−0.176	−0.150	−0.078	−0.066	−0.042	−0.045	−0.014	0.005	0.030
0.5	−0.056	0.099	−0.054	−0.037	−0.033	−0.026	−0.019	−0.009	−0.007	−0.005
0.7	−0.009	−0.059	0.035	−0.012	−0.013	−0.005	−0.002	−0.009	−0.002	0.006
0.9	−0.031	−0.031	−0.009	0.012	0.002	0.013	0.021	0.015	0.008	0.004
1	0.014	−0.023	0.000	0.011	0.009	0.019	0.022	0.020	0.018	0.004
1.3	0.002	−0.004	0.022	0.034	0.027	0.030	0.084	0.034	0.035	0.028
1.5	0.009	0.023	0.038	0.044	0.037	0.042	0.034	0.174	0.040	0.032
1.7	0.028	0.029	0.049	0.054	0.047	0.050	0.047	0.036	0.277	0.039
2	0.040	0.051	0.065	0.068	0.059	0.063	0.060	0.051	0.041	0.464
n=100										
0.1	−0.028	−0.216	−0.175	−0.113	−0.103	−0.063	0.036	0.169	0.452	0.349
0.3	1.874	−0.135	−0.125	−0.052	−0.044	−0.022	−0.038	−0.023	0.009	0.024
0.5	−0.002	0.146	−0.034	−0.026	−0.025	−0.021	−0.019	-0.001	−0.008	−0.009
0.7	0.000	−0.042	0.045	−0.009	−0.013	−0.009	0.000	−0.009	−0.008	−0.001
0.9	0.007	−0.025	−0.015	0.001	−0.004	0.005	0.009	0.013	−0.001	−0.003
1	0.014	−0.010	−0.007	−0.001	−0.001	0.005	0.009	0.014	0.010	0.009
1.3	0.036	0.010	0.006	0.015	0.010	0.010	0.065	0.012	0.019	0.014
1.5	0.041	0.023	0.018	0.022	0.017	0.018	0.006	0.158	0.016	0.015
1.7	0.052	0.027	0.028	0.032	0.024	0.025	0.016	0.009	0.267	0.019
2	0.056	0.043	0.042	0.043	0.033	0.034	0.023	0.020	0.013	0.454

**Table 2:** MSE of the MREE for different α, β and sample sizes *n* under pure data.

β	α									
	0.3	0.5	0.7	0.9	1	1.1	1.3	1.5	1.7	2
n=20										
0.1	0.347	0.251	0.222	0.145	0.122	0.106	0.098	0.242	0.206	0.240
0.3	7.506	0.147	0.100	0.069	0.063	0.059	0.059	0.062	0.098	0.169
0.5	0.238	0.076	0.067	0.051	0.049	0.047	0.050	0.055	0.064	0.101
0.7	0.177	0.091	0.056	0.045	0.044	0.043	0.045	0.055	0.056	0.071
0.9	0.163	0.085	0.061	0.045	0.042	0.043	0.047	0.053	0.058	0.064
1	0.171	0.085	0.064	0.045	0.042	0.045	0.048	0.053	0.058	0.063
1.3	0.148	0.082	0.065	0.052	0.046	0.046	0.061	0.055	0.058	0.065
1.5	0.146	0.085	0.069	0.056	0.050	0.050	0.051	0.087	0.061	0.065
1.7	0.150	0.085	0.070	0.060	0.053	0.055	0.055	0.056	0.134	0.066
2	0.132	0.091	0.076	0.065	0.059	0.060	0.060	0.060	0.061	0.265
n=50										
0.1	0.334	0.170	0.118	0.066	0.044	0.037	0.067	0.195	0.401	0.275
0.3	5.050	0.093	0.051	0.026	0.021	0.020	0.024	0.027	0.035	0.050
0.5	0.196	0.059	0.030	0.018	0.017	0.018	0.021	0.026	0.030	0.037
0.7	0.191	0.053	0.031	0.018	0.016	0.017	0.023	0.025	0.028	0.035
0.9	0.131	0.050	0.029	0.019	0.016	0.018	0.022	0.025	0.028	0.029
1	0.154	0.044	0.031	0.018	0.017	0.020	0.022	0.024	0.027	0.031
1.3	0.112	0.046	0.029	0.023	0.018	0.018	0.033	0.028	0.029	0.031
1.5	0.108	0.049	0.033	0.024	0.020	0.022	0.022	0.059	0.031	0.031
1.7	0.119	0.049	0.036	0.026	0.022	0.023	0.025	0.025	0.108	0.033
2	0.108	0.053	0.040	0.030	0.025	0.026	0.028	0.029	0.028	0.249
n=100										
0.1	0.295	0.139	0.085	0.038	0.022	0.022	0.068	0.201	0.583	0.403
0.3	4.770	0.075	0.039	0.016	0.011	0.011	0.017	0.019	0.023	0.035
0.5	0.189	0.061	0.022	0.011	0.009	0.012	0.016	0.017	0.022	0.023
0.7	0.141	0.038	0.024	0.010	0.009	0.010	0.014	0.017	0.018	0.021
0.9	0.123	0.035	0.021	0.011	0.009	0.011	0.012	0.015	0.019	0.021
1	0.122	0.036	0.019	0.010	0.009	0.011	0.013	0.016	0.017	0.020
1.3	0.114	0.035	0.019	0.012	0.009	0.010	0.021	0.016	0.017	0.019
1.5	0.105	0.037	0.019	0.012	0.010	0.011	0.012	0.045	0.017	0.020
1.7	0.097	0.034	0.021	0.014	0.011	0.012	0.014	0.014	0.092	0.020
2	0.088	0.039	0.023	0.016	0.012	0.013	0.013	0.016	0.016	0.227

**Table 3:** Bias of the MREE for different α, β and sample sizes *n* under contaminated data.

β	α									
	0.3	0.5	0.7	0.9	1	1.1	1.3	1.5	1.7	2
n=20										
0.1	−0.104	−0.382	−0.340	−0.243	−0.131	−0.071	0.090	0.188	0.295	0.379
0.3	3.287	−0.157	−0.187	−0.135	−0.113	−0.091	−0.045	0.013	0.107	0.237
0.5	2.691	1.483	−0.024	−0.067	−0.069	−0.043	−0.031	−0.010	−0.003	0.051
0.7	3.004	2.546	1.168	0.036	−0.017	−0.008	0.003	0.006	0.005	0.010
0.9	3.133	2.889	2.319	0.917	0.222	0.058	0.019	0.023	0.017	0.022
1	3.183	2.986	2.558	1.619	0.805	0.214	0.039	0.030	0.031	0.019
1.3	3.239	3.121	2.902	2.550	2.262	1.872	0.613	0.077	0.049	0.040
1.5	3.255	3.170	3.012	2.775	2.606	2.396	1.676	0.571	0.069	0.051
1.7	3.271	3.194	3.071	2.903	2.790	2.661	2.256	1.489	0.578	0.057
2	3.289	3.216	3.122	3.012	2.942	2.865	2.649	2.305	1.690	0.682
n=50										
0.1	0.384	−0.170	−0.189	−0.132	−0.054	0.024	0.104	0.171	0.261	0.382
0.3	3.549	0.000	−0.122	−0.086	−0.077	−0.053	−0.023	0.029	0.054	0.118
0.5	2.875	1.771	0.040	−0.048	−0.048	−0.029	−0.013	−0.015	−0.017	0.003
0.7	3.091	2.698	1.294	0.048	−0.010	−0.014	−0.001	0.004	0.001	−0.005
0.9	3.205	2.945	2.379	0.939	0.226	0.045	0.009	0.013	0.012	0.013
1	3.240	3.011	2.612	1.609	0.793	0.196	0.018	0.014	0.021	0.012
1.3	3.316	3.171	2.925	2.548	2.239	1.819	0.554	0.034	0.020	0.020
1.5	3.346	3.223	3.034	2.780	2.596	2.363	1.589	0.502	0.035	0.022
1.7	3.362	3.254	3.100	2.916	2.791	2.643	2.199	1.383	0.518	0.025
2	3.373	3.281	3.162	3.035	2.955	2.865	2.622	2.236	1.575	0.650
n=100										
0.1	0.610	−0.138	−0.105	−0.031	0.002	0.040	0.117	0.184	0.270	0.381
0.3	3.906	0.136	−0.071	−0.050	−0.052	−0.028	−0.028	−0.008	0.023	0.066
0.5	2.927	1.934	0.101	−0.034	−0.027	−0.016	0.006	0.000	−0.003	−0.008
0.7	3.122	2.761	1.348	0.066	0.004	−0.007	0.007	0.011	0.012	0.000
0.9	3.241	2.955	2.406	0.958	0.238	0.047	0.004	0.014	0.022	0.017
1	3.289	3.045	2.651	1.622	0.798	0.202	0.010	0.011	0.016	0.023
1.3	3.362	3.204	2.944	2.567	2.245	1.812	0.533	0.028	0.015	0.022
1.5	3.384	3.269	3.058	2.802	2.610	2.369	1.567	0.485	0.027	0.018
1.7	3.405	3.305	3.133	2.940	2.811	2.658	2.196	1.357	0.504	0.018
2	3.421	3.327	3.204	3.065	2.980	2.886	2.633	2.234	1.541	0.637

**Table 4:** MSE of the MREE for different α, β and sample sizes *n* under contaminated data.

β	α									
	0.3	0.5	0.7	0.9	1	1.1	1.3	1.5	1.7	2
n=20										
0.1	0.403	0.248	0.465	0.576	1.025	1.093	1.613	1.565	1.626	1.591
0.3	12.595	0.142	0.103	0.075	0.192	0.188	0.362	0.590	1.016	1.537
0.5	7.443	2.268	0.088	0.062	0.058	0.059	0.065	0.189	0.241	0.527
0.7	9.209	6.645	1.410	0.069	0.056	0.058	0.063	0.068	0.119	0.208
0.9	9.982	8.493	5.512	0.882	0.119	0.068	0.065	0.069	0.075	0.090
1	10.292	9.072	6.672	2.692	0.693	0.117	0.068	0.070	0.076	0.087
1.3	10.664	9.916	8.574	6.641	5.240	3.610	0.430	0.079	0.079	0.089
1.5	10.778	10.229	9.238	7.850	6.940	5.883	2.917	0.389	0.079	0.087
1.7	10.884	10.379	9.599	8.582	7.942	7.234	5.235	2.326	0.403	0.087
2	11.004	10.515	9.915	9.233	8.814	8.369	7.177	5.472	2.998	0.547
n=50										
0.1	1.552	0.815	0.741	0.703	0.966	1.190	1.129	1.224	1.165	1.210
0.3	14.969	0.105	0.047	0.030	0.078	0.075	0.280	0.559	0.566	0.881
0.5	8.345	3.190	0.049	0.025	0.021	0.022	0.025	0.029	0.035	0.184
0.7	9.634	7.335	1.694	0.031	0.020	0.022	0.027	0.029	0.033	0.039
0.9	10.353	8.723	5.712	0.898	0.077	0.027	0.028	0.030	0.032	0.039
1	10.578	9.126	6.871	2.619	0.645	0.067	0.027	0.030	0.033	0.039
1.3	11.069	10.129	8.608	6.548	5.064	3.359	0.329	0.033	0.034	0.038
1.5	11.263	10.457	9.268	7.787	6.801	5.648	2.576	0.279	0.032	0.038
1.7	11.371	10.655	9.676	8.567	7.854	7.051	4.908	1.968	0.298	0.037
2	11.449	10.833	10.060	9.275	8.793	8.276	6.947	5.079	2.560	0.461
n=100										
0.1	2.102	0.399	0.808	0.945	0.924	0.929	0.891	1.012	1.233	1.120
0.3	17.185	0.141	0.033	0.018	0.013	0.014	0.018	0.142	0.258	0.453
0.5	8.624	3.768	0.056	0.015	0.011	0.015	0.017	0.018	0.022	0.028
0.7	9.809	7.646	1.828	0.024	0.011	0.013	0.018	0.019	0.020	0.023
0.9	10.559	8.764	5.812	0.927	0.070	0.018	0.017	0.020	0.021	0.023
1	10.870	9.312	7.058	2.648	0.645	0.057	0.017	0.019	0.021	0.023
1.3	11.342	10.306	8.691	6.619	5.068	3.312	0.297	0.020	0.020	0.023
1.5	11.494	10.727	9.379	7.880	6.845	5.646	2.484	0.251	0.021	0.021
1.7	11.632	10.960	9.848	8.675	7.932	7.101	4.866	1.873	0.272	0.022
2	11.739	11.102	10.297	9.422	8.910	8.363	6.973	5.040	2.420	0.430

## Abbreviations

The following abbreviations are used in this manuscript:

KLD	Kullback-Leibler Divergence
LDPD	Logarithmic Density Power Divergence
LSD	Logarithmic Super Divergence
GRE	Generalized Renyi Entropy
MRE	Minimum Relative (α,β)-entropy
MREE	Minimum Relative (α,β)-entropy Estimator
MSE	Mean Squared Error
